# On-Site Ribosome Remodeling by Locally Synthesized Ribosomal Proteins in Axons

**DOI:** 10.1016/j.celrep.2019.11.025

**Published:** 2019-12-10

**Authors:** Toshiaki Shigeoka, Max Koppers, Hovy Ho-Wai Wong, Julie Qiaojin Lin, Roberta Cagnetta, Asha Dwivedy, Janaina de Freitas Nascimento, Francesca W. van Tartwijk, Florian Ströhl, Jean-Michel Cioni, Julia Schaeffer, Mark Carrington, Clemens F. Kaminski, Hosung Jung, William A. Harris, Christine E. Holt

**Affiliations:** 1 Department of Physiology, Development and Neuroscience, University of Cambridge, Downing Street, Cambridge CB2 3DY, UK; 2 Department of Biochemistry, University of Cambridge, Cambridge CB2 1GA, UK; 3 Department of Chemical Engineering and Biotechnology, University of Cambridge, Cambridge CB3 0AS, UK

**Keywords:** axon, mRNA, local translation, ribosome, ribosomal proteins, Rps4x, axonal protein synthesis, ribosome remodeling, axon branching, neural wiring

## Abstract

Ribosome assembly occurs mainly in the nucleolus, yet recent studies have revealed robust enrichment and translation of mRNAs encoding many ribosomal proteins (RPs) in axons, far away from neuronal cell bodies. Here, we report a physical and functional interaction between locally synthesized RPs and ribosomes in the axon. We show that axonal RP translation is regulated through a sequence motif, CUIC, that forms an RNA-loop structure in the region immediately upstream of the initiation codon. Using imaging and subcellular proteomics techniques, we show that RPs synthesized in axons join axonal ribosomes in a nucleolus-independent fashion. Inhibition of axonal CUIC-regulated RP translation decreases local translation activity and reduces axon branching in the developing brain, revealing the physiological relevance of axonal RP synthesis *in vivo*. These results suggest that axonal translation supplies cytoplasmic RPs to maintain/modify local ribosomal function far from the nucleolus in neurons.

## Highlights

• Axonal ribosomal protein (RP) synthesis is cue regulated via a 5′ UTR loop-forming motif
• Axonal RP synthesis generates a free cytosolic pool of ribosomal proteins in axons
• Axonally synthesized RPs join axonal ribosomes in a nucleolus-independent manner
• Local RP synthesis is required for ribosome function and branch architecture

## Graphical Abstract

**Figure:**
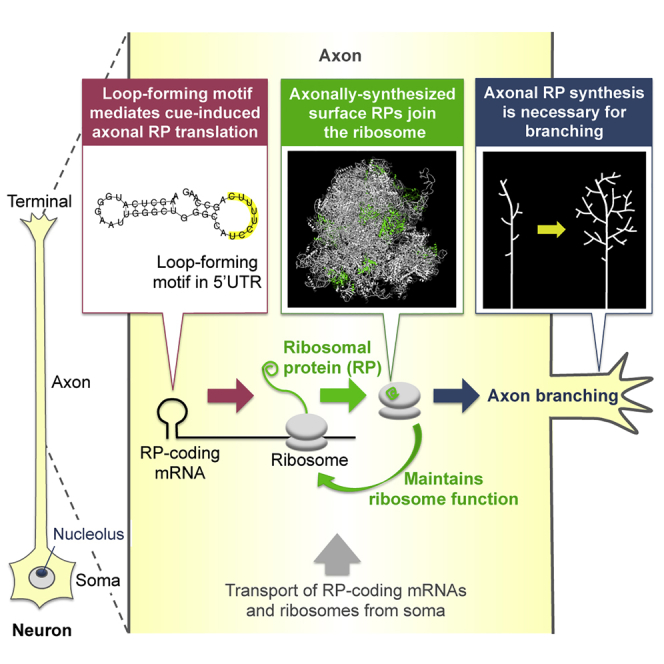

## Teaser

Local protein synthesis in axons supplies new ribosomal proteins far from the nucleolus, the known site of ribosome biogenesis. Shigeoka et al. provide evidence that axonally synthesized ribosomal proteins join pre-existing ribosomes and maintain translation activity in axons, which is required for axon terminal branching.

## Introduction

RNA localization and local translation play key roles in the assembly, maintenance, and repair of neuronal connections (Deglincerti and Jaffrey, 2012, Holt and Schuman, 2013, Jung et al., 2012, Rishal and Fainzilber, 2014, Willis and Twiss, 2006). Recent genome-wide studies on the axonal transcriptome reveal that thousands of mRNAs are localized to axons (Andreassi et al., 2010, Gumy et al., 2011, Nijssen et al., 2018, Zivraj et al., 2010). A consistent but unexpected finding of these studies is the robust enrichment of mRNAs that encode ribosomal proteins (RPs) in axons of a variety of neuron types (Andreassi et al., 2010, Briese et al., 2016, Cajigas et al., 2012, Gioio et al., 2004, Gumy et al., 2011, Moccia et al., 2003, Saal et al., 2014, Taylor et al., 2009, Zivraj et al., 2010). This finding cannot simply be explained by Brownian diffusion of mRNAs from the soma, since several studies showed that these transcripts are significantly enriched in the axon compared to the cell body (Andreassi et al., 2010, Saal et al., 2014), suggesting the presence of mechanisms that selectively target RP-coding transcripts to the axon. Furthermore, recent studies provide evidence that RP-coding mRNAs are robustly translated in retinal ganglion cell (RGC) axons both *in vivo* (Shigeoka et al., 2016) and *in vitro* (Cagnetta et al., 2018), raising the possibility that locally supplied RPs serve to support axonal function.

Axons are long neuronal processes that carry out many vital specific cellular functions far from their cell bodies, including translation, and must therefore maintain their protein synthetic machinery in good order. However, because eukaryotic ribosome assembly is known to occur mainly in the nucleolus (Fromont-Racine et al., 2003, Lastick and McConkey, 1976, Peña et al., 2017), the physiological function of axonally synthesized RPs in a neuronal subcellular compartment far distant from the nucleus is enigmatic. Recent studies on spinal muscular atrophy (SMA) implicated a potential role for free RPs in the maintenance of ribosomes in axons (Bernabò et al., 2017, Rage et al., 2013). The depletion of the survival motor neuron (SMN) protein, an RNA-binding protein that associates with RP-coding mRNAs (Rage et al., 2013), caused a significant decrease in translation levels of RP-coding mRNAs (Bernabò et al., 2017). SMN depletion also leads to a 27% reduction in the number of ribosomes in axons (Bernabò et al., 2017). Although the causal relation remains uncertain, one possibility is that axonally synthesized RPs are used to make integral components of functional ribosomes.

The eukaryotic ribosome is a macromolecular machine composed of 4 ribosomal RNA (rRNA) molecules and ∼80 different RPs. Eukaryotic RPs are shipped into the nucleus for assembly into ribosomal subunits within the nucleolus, although a few ribosomal proteins are added to the ribosome in the cytoplasm, such as Rpl24/eL24, Rpl10/uL16, and Rplp0/uL10 (Panse and Johnson, 2010). A number of previous studies called into question the widely held view of the ribosome as a stable molecular machine whose components remain unchangeable during its lifetime. For example, several RPs in the ribosome have higher turnover rates than other RP components, suggesting the possibility that individual RPs in the ribosome can be replaced by free cytoplasmic RPs (Lastick and McConkey, 1976, Mathis et al., 2017, Samir et al., 2018). These studies, together with the robust axonal translation of RPs, prompted us to ask whether axonal ribosomes incorporate locally synthesized RPs to maintain ribosome function far from the cell body.

In this study, we explored roles of axonally synthesized RPs using a range of technical approaches, including live imaging, *in vivo* axon-specific knockdown, and mass spectrometry-based proteomics. We found that RP translation is regulated by a branch-promoting factor, Netrin-1, through a loop structure-forming sequence motif called CUIC, that is shared by ∼70% of RP-coding mRNAs. Isoforms of RP mRNAs with a short 5′ UTR truncated at the CUIC region are highly enriched in axons. Transcriptome and proteome analyses revealed that the structural positions of axonally abundant RPs are biased toward the surface of the ribosome subunits. Live imaging and subcellular proteomic approaches showed that axonally synthesized RPs physically associate with axonal ribosomes in a nucleolus-independent fashion. Furthermore, we show that inhibition of axonal RP synthesis leads to a significant decrease in the level of axonal mRNA translation and severe axon branching defects *in vivo*. These results support the view that ribosome function is maintained by a cytoplasmic pool of locally synthesized RPs in axons.

## Results

### RP-Coding mRNAs Harbor a Common Loop Structure-Forming Sequence Motif in Their 5′ UTR

**Figure 1:**
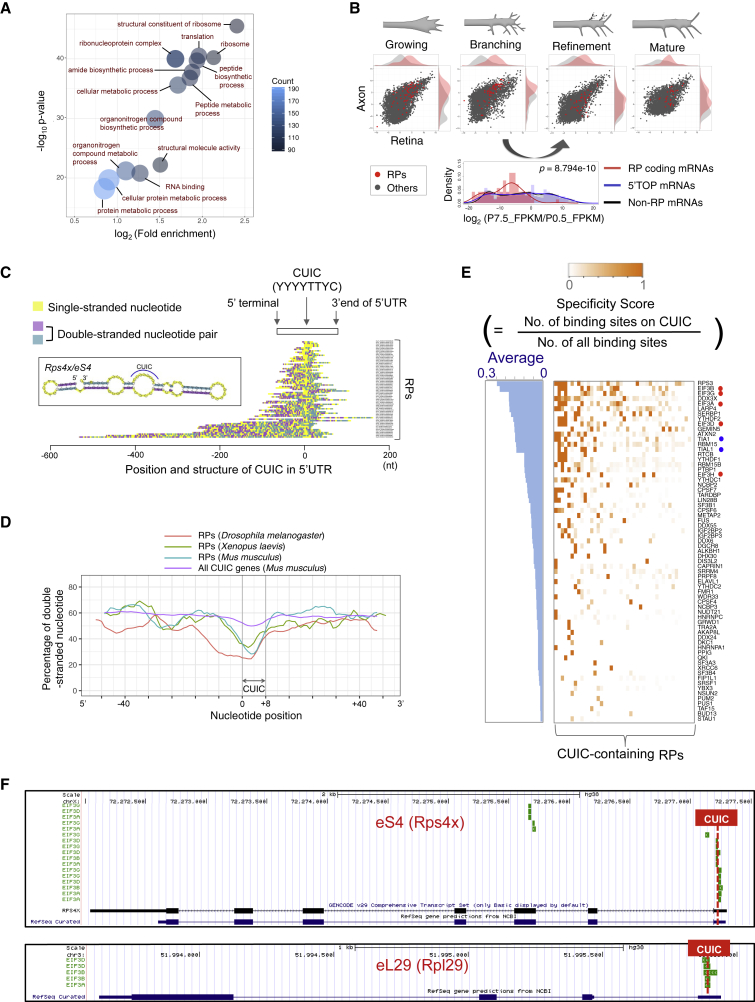
RP-Coding mRNAs Harbor a Common Loop Structure-Forming Sequence Motif in the 5′ UTR (A) Enrichment of GO terms in the *Xenopus laevis* RGC axon transcriptome. (B) Relative abundance (FPKM) of translated mRNAs coding for RPs (red) and other proteins (gray) in the mouse RGC axon (y axis) and retina (x axis), obtained by the Axon-TRAP system *in vivo*. The histogram (lower) shows the distribution of the ratio of abundance of 2 consecutive stages (refinement [P7.5]/branching [P0.5]). p value: Kolmogorov-Smirnov test between RP coding mRNAs and non-RP mRNAs. (C) Relative position of the CUIC and RNA-secondary structure of 5′ UTRs of mouse RP-coding mRNAs. Each bar represents 5′ UTR sequences and is colored by the predicted secondary structure. The position of CUIC is aligned at center (0 nt) and the x axis indicates the distance from the CUIC motif. (D) Average fraction of double-stranded nucleotides around the CUIC motif in 5′ UTRs of all CUIC-containing mouse genes and RPs (moving average, 7-nt window). (E) The ranking of RBPs that specifically bind to the CUIC motif of RP-coding mRNAs. The heatmap color indicates the specificity score calculated by the formula in the upper panel. The histogram (left) shows the average of the specificity score of each RBP for all CUIC-containing RP mRNAs. Red dots mark eIF3 components, and blue dots mark TIA1 and TIAL1. (F) UCSC Genome browser view of CLIP clusters of eIF3 components on 2 RP-coding mRNAs.

To analyze the axonal transcriptome and proteome, we used a well-established method to harvest pure RGC axons from embryonic *Xenopus* eyes grown on microporous transfilters (Figure S1A) (Cagnetta et al., 2018, Zheng et al., 2001). The intact nature of the eyes permits only the axons, not the dendrites, of RGCs to grow out of the eye (via the optic nerve) onto the transfilter with soma-excluding 1μm pores (Figure S1A). The purity of the axon sample was confirmed by RT-PCR and immunostaining of soma/nuclear factors (Figure S1B) (Cagnetta et al., 2018). Using this pure axon material, we first performed an RNA sequencing (RNA-seq) analysis of RGC axons. Consistent with previous studies (Deglincerti and Jaffrey, 2012), RPs were robustly enriched in the axonal transcriptome (Figure 1A). Next, we investigated a potential physiological role of axonal RP synthesis by analyzing our genome-wide translatome data of mouse RGC axons *in vivo* (Shigeoka et al., 2016). The axonal translation of most RP-coding mRNAs peaks during the branching/synaptogenesis stage (postnatal day [P] 0.5) and declines thereafter in a synchronous manner (Figure 1B). The pattern of translational changes of RP-coding mRNAs during the postnatal period (P0.5–P7.5) was significantly different from the other translated mRNAs (Figure 1B, lower panel), suggesting the presence of a mechanism that co-regulates the synthesis of many different RPs in axons.

The coordinated axonal RP synthesis led us to infer that RP-coding mRNAs may have a common *cis*-regulatory element(s). A *de novo* motif discovery algorithm (Heinz et al., 2010) revealed that ∼70% of RP-coding mRNAs share a 5′ UTR sequence motif, YYYYTTYC (Figure S1C). Since this motif is located immediately (20–80 nt) upstream of the initiation codon of RP-coding mRNAs in most cases (>90%) (Figures 1C and S1D), we called this motif *cis*-element upstream of the initiation codon (CUIC). Gene Ontology (GO) enrichment analysis revealed that not only ribosome-related GO terms but also those linked to neuron morphogenesis are enriched in all CUIC-containing mouse genes (Figure S1E), suggesting a potential role of the CUIC motif in axon projection and branching. Consistent with this, translatome analysis in RGC axons reveals that CUIC-containing transcripts have significantly higher translation levels than those without (Figure S1F). The predicted RNA secondary structure of 5′ UTRs of RP-coding mRNAs shows that the nucleotide region around the CUIC motif tends to form a single-stranded loop, which may allow *trans* factors to recognize this motif (Figures 1C and S1G; Table S1). The sequence and the loop structure are well conserved among animal species (Figures 1D and S1H).

To explore which RNA-binding proteins (RBPs) associate with the CUIC motif, we analyzed published cross-linking immunoprecipitation (CLIP)-seq datasets in the POSTAR2 database (Zhu et al., 2019). In this analysis, we counted the number of CLIP clusters that overlapped with the CUIC region of RP-coding mRNAs for each RBP and normalized them to the total number of clusters on any region of the mRNAs to evaluate the specificity of RBP binding (Figure 1E). This revealed that components of the eIF3 complex are particularly enriched in the proteins that specifically bind to the CUIC motif of RP-coding mRNAs (Figures 1E and 1F).

### CUIC Motif Is Associated with Alternative 5′ Ends and Netrin-1-Stimulated Translation of Axonal mRNAs

**Figure 2:**
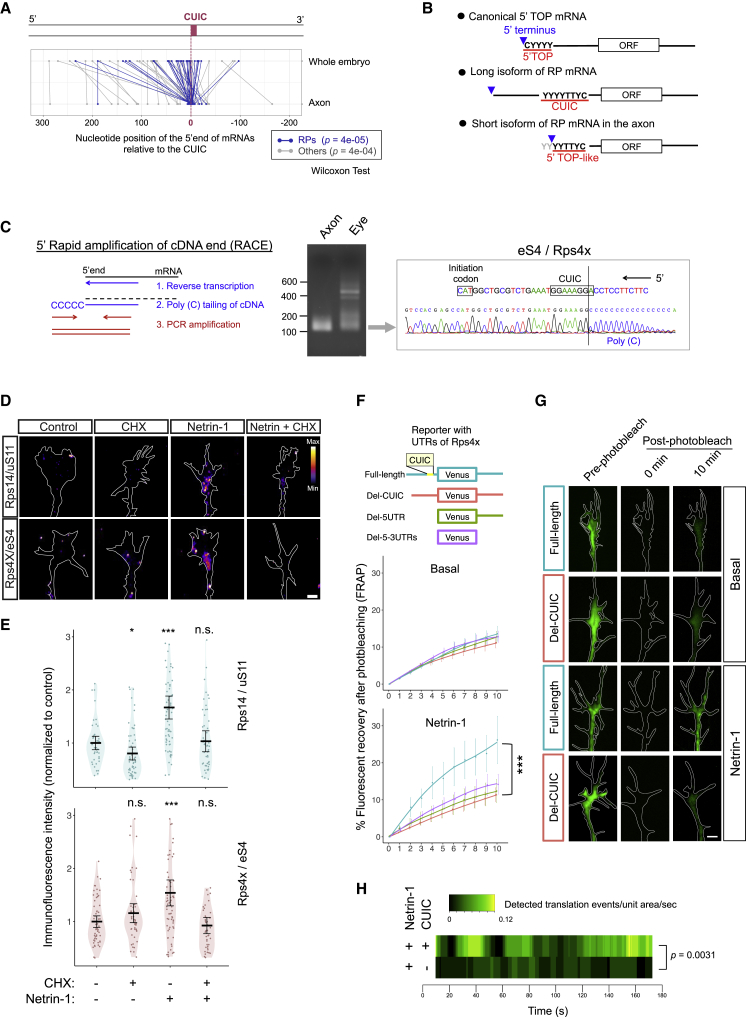
CUIC Motif Is Involved with Alternative 5′ End and Netrin-1-Stimulated Translation of Axonal RP mRNAs (A) Plot showing the difference of the position of 5′ terminal of CUIC-containing mRNAs between the axon and the whole embryo. The x axis indicates the relative position of 5′ terminal compared to CUIC. (B) Diagram showing 2 isoforms of the CUIC-containing mRNA with alternative 5′ ends and the 5′ TOP mRNA. (C) Diagram (left), gel image (middle), and Sanger sequencing result (right) of 5′ RACE products of Rps4x/eS4 mRNAs in axon and eye samples. (D and E) Representative images (D) and quantification of quantitative immunofluorescence (QIF) (E) for Rps14/uS11 (n = 42, control; 59, CHX; 66, Netrin-1; 64, Netrin-1 + CHX) and Rps4x/eS4 (n = 59, control; 50, CHX; 70, Netrin-1; 44, Netrin-1 + CHX) in RGC growth cones with or without Netrin-1 (5 min)/cycloheximide (CHX) treatment (bars, average with 95% confidence interval [CI], Mann-Whitney *U* test compared to the control, ^∗∗∗^p ≤ 0.001, ^∗^p ≤ 0.05). Scale bar, 5 μm. (F and G) Plots (F) and representative images (G) of relative fluorescence recovery of Venus reporter constructs after photobleaching (error bar, SEM) in RGC growth cones. ^∗∗∗^p < 0.0001; 2-way ANOVA comparing full-length UTRs (n = 8) with Del-motif (n = 12). Scale bar, 2 μm. (H) Moving average (20 s window) of the count of detected translation events per unit area per second with the Netrin-1 stimulation in single-molecule translation imaging. p value: Mann-Whitney *U* test between full-length UTRs (n = 11) and Del-motif (n = 12) using the total count in each growth cone.

Our analysis of CLIP-seq data also showed that TIA1 and TIAL1 bind with high specificity to the CUIC region (Figure 1E). Since these proteins were reported to be key factors in 5′ terminal oligopyrimidine tract (5′ TOP) mRNA regulation (Damgaard and Lykke-Andersen, 2011), we sought to understand the relation between CUIC and 5′ TOP mRNAs. Although both the CUIC and 5′ TOP motif are a tract of pyrimidine nucleotides in the 5′ UTR, CUIC is always immediately upstream of the initiation codon and is usually distant from the 5′ end, unlike the 5′ TOP sequence, which is always at the 5′ terminal of mRNAs (Meyuhas, 2000). Several RP-coding mRNAs contain both the 5′ TOP and CUIC motifs in separate positions (Figure S2A). However, since we found that the mouse expressed sequence tag (EST) showed that the positions of 5′ ends of the CUIC-containing RP mRNAs are highly variable, an interesting possibility is that the alternative 5′ end formed at the CUIC region generates a 5′ TOP-like sequence. To explore this possibility, we analyzed the 5′ end sequence of CUIC-containing mRNAs in our *Xenopus* RGC axon RNA-seq data. Whereas the 5′ ends of CUIC-containing transcripts detected in *Xenopus* whole embryos (Ding et al., 2017) are located upstream (0–300 nt) of the CUIC region in most cases, the 5′ ends of those in the RGC axon sample tended to have short 5′ UTRs that were truncated at the CUIC region (Figures 2A, 2B, and S2B). This prevalence of short isoforms in axons was validated by 5′ rapid amplification of cDNA ends (RACE) comparing the 5′ end of Rps4x/eS4 transcripts between the RGC axon and the somal (whole-eye) samples (Figure 2C). Sequencing of the amplified cDNAs showed that the 5′ end of the Rps4x/eS4 short isoform is precisely located at the CUIC region (Figure 2C). These results suggest that the alternative 5′ end at the CUIC region can generate a 5′ TOP-like sequence in RP-coding mRNAs (Figure 2B). Since 5′ TOP mRNAs are regulated by the mammalian target of rapamycin (mTOR) pathway (Jefferies et al., 1997), and the mTOR pathway is required for Netrin-1-mediated local translation (Campbell and Holt, 2001), the CUIC motif could play a crucial role in regulated RP synthesis in the axon. To investigate the relation between Netrin-1 stimulation and CUIC-containing RP mRNAs, we analyzed a proteomics dataset of the cue-induced nascent (newly synthesized) proteome in cultured *Xenopus* RGC axons (Cagnetta et al., 2018). The analysis revealed that RPs are particularly enriched in the group of proteins whose translation is promoted by Netrin-1 (p = 0.0001, enrichment of GO: 0005840) (Figure S2C; Tables S2 and S3). Consistent with this result, quantification of the immunofluorescence (QIF) signal showed that Netrin-1 increases the translation of most, but not all, RPs tested in axonal growth cones (Figures 2D, 2E, S2D, and S2E). We next investigated the effect of the CUIC motif on axonal RP synthesis by performing fluorescence recovery after photobleaching (FRAP) experiments using the fast-folding fluorescent protein Venus, expressed from mRNAs with and without the CUIC motif in the UTR. We tested the UTR of Rps4x/eS4, the RP that shows the greatest increase in axonal translation after Netrin-1 stimulation (Figures 2D, 2E, and S2C). While no significant difference was observed in the FRAP signal in basal conditions over the 10-min period of imaging, the addition of Netrin-1 elicited a significantly higher FRAP signal with the full-length 5′ UTR reporter compared to the CUIC-deleted or 5′ UTR-deleted reporter constructs (Figures 2F and 2G), indicating a higher translation rate of the CUIC-containing mRNA. The inhibition of translation with anisomycin abolished this difference (Figure S2F). Consistent with these FRAP results, independent experiments using a single-molecule translational imaging approach (Ifrim et al., 2015, Ströhl et al., 2017, Tatavarty et al., 2012) that captures individual translation events in real-time revealed a significantly higher number of translation events with the CUIC-containing reporter, compared to the CUIC-deleted reporter, in Netrin-1-stimulated axonal growth cones (Figure 2H). These data provide evidence that the CUIC motif is, at least partially, responsible for the Netrin-1-induced axonal translation of RPs.

### Surface Components of the Ribosomal Subunits Are Enriched in the Axoplasm

The coordinated regulation of numerous axonally synthesized RPs suggests that they may have a common ribosome-related function rather than a disparate variety of extra-ribosomal roles (Warner and McIntosh, 2009) in axons. However, since most ribosomal proteins are thought to be assembled into ribosomes exclusively in the nucleolus, it seemed puzzling that ribosomal proteins are synthesized in the distal axon, far away from the nucleolus (Fromont-Racine et al., 2003). To test the possibility that the retrograde transport of axonally synthesized RPs allows their assembly into ribosomes in the nucleolus, we focused on the abundance of RPs outside of ribosomes (free cytoplasmic RPs) in the distal axon. Robust axonal RP translation should result in the accumulation of free RPs in the axon, unless these are retrogradely transported toward the soma or quickly degraded. To detect free RPs in the axon, we performed sucrose density gradient fractionation on pure-axon lysates, generated using Boyden chambers, as well as on whole-brain lysates (Figures S3A–S3C). Mass spectrometry analysis of the fractionated samples showed a robust accumulation of free RPs in ribosome-free fractions of the axon lysate, whereas RPs were strongly depleted in ribosome-free fractions of the whole-brain lysate (Figure S3D), suggesting that axonally translated RPs have function(s) in the axon rather than in the nucleolus.

**Figure 3:**
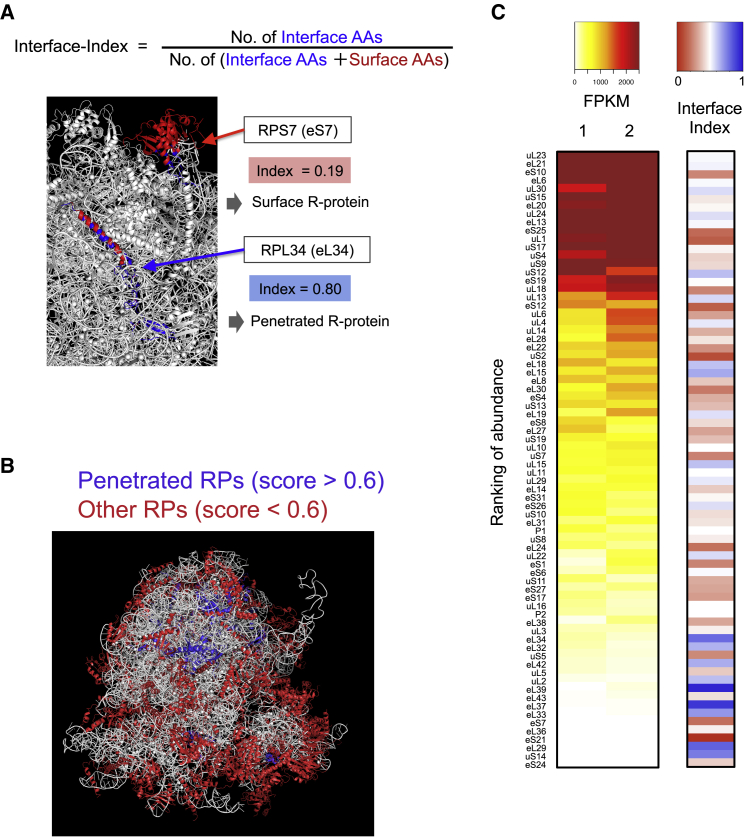
Structural Positions of RPs Encoded by Axonally Localized mRNAs Are Surface Biased (A) Formula of interface-index (upper) and a partial structure of the ribosome, generated with PyMOL, showing the relation between RP position and interface-indices. (B) Human 80S ribosome structure, generated with PyMOL, showing the position of all RPs that are classified based on the interface-index. (C) Ranking of abundance (average FPKM) of RPs coding mRNAs (n = 2) and interface-index scores of RPs (more blue = higher, more red = lower).

To evaluate whether axonally translated RPs are involved in ribosomal function, we focused on the position of these RPs in the ribosome structure. We reasoned that if the axonally translated RPs are used to replace/repair components of the ribosome, the position of these RPs may be biased to the surface of the ribosome because RPs that penetrate deeply into the rRNA core of ribosomal subunits are less likely to be replaced or detached. To address this, we performed an unbiased classification of RPs (surface or core/penetrated) based on their structural position in the human 80S ribosome structure (Natchiar et al., 2017). For each RP, we assigned an interface-index score based on the fraction of residues that interface with rRNA by using the PyMOL function InterfaceResidues (https://pymolwiki.org/index.php/Main_Page). RPs with a high interface-index score (high fraction of rRNA interface residues) tend to be located deep in the ribosome core or have long tails that penetrate into the core (Figures 3A and 3B). We found that the group of RPs whose mRNAs are abundant in the axon (FPKM > 100, 61 RPs) had a significantly lower interface-index average (0.42) than the less abundant (FPKM < 100) group (0.57, Kolmogorov-Smirnov test, p = 0.014) (Figure 3C). Consistently, RPs with high interface-indices (>0.6; Figure 3B) were significantly depleted from the abundant group (5.0-fold depletion, Fisher’s exact test, p = 0.0016) (Figure 3C). Although label-free quantification (LFQ) of mass spectrometry is a less robust quantitative method compared to RNA-seq, we observed a similar trend in which high interface-indices (>0.6) were significantly depleted from the group of RPs with high abundance (LFQ abundance score > 1) in the axonal ribosome-free fraction (Figure S3E). These results indicate that the axon, but not the cell body, contains a large cytoplasmic pool of extra-ribosomal RPs that are biased to occupy superficial structural positions in the ribosome and suggest the possibility that surface components of axonal ribosomes may be replaced by these cytoplasmic free RPs in axons.

### Axonally Synthesized RPs Become Physically Associated with Ribosomes

**Figure 4:**
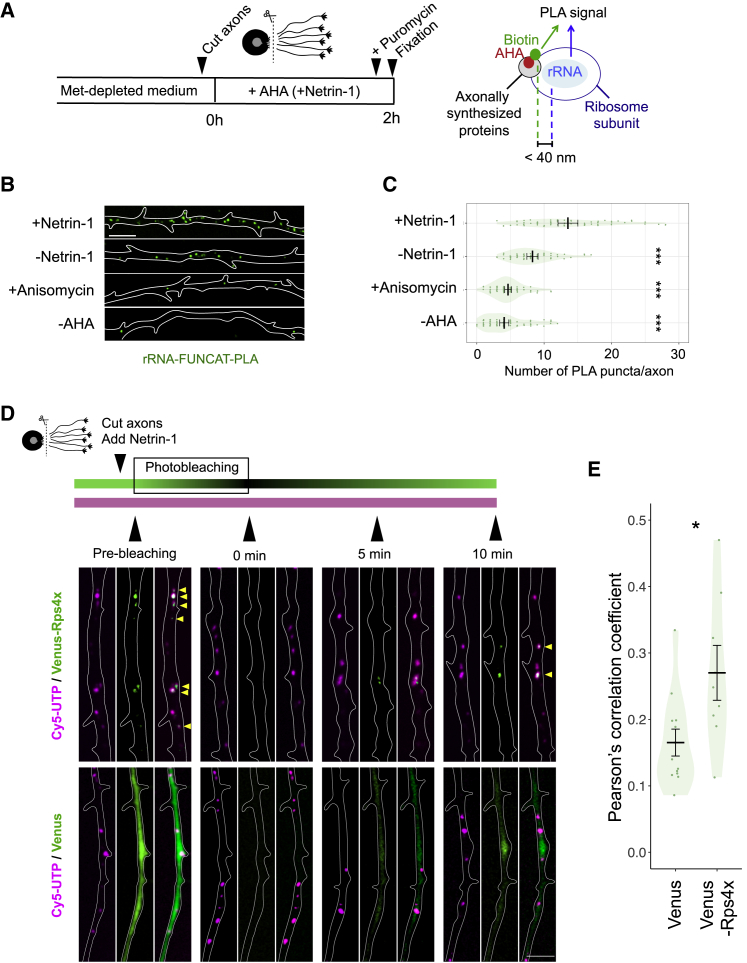
Axonally Synthesized RPs Co-localize with Ribosome-Containing Granules (A) Experimental workflow and a diagram describing the positional relation of each protein/probe in the rRNA-FUNCAT-PLA experiments. (B and C) Representative images (B) and plots for the number of PLA puncta in each condition (C) detected in the axon (bars, average with 95% CI, Mann-Whitney *U* test, ^∗∗∗^p ≤ 0.001). Scale bar, 5 μm. (D) Experimental workflow of the Venus + Cy5-UTP FRAP experiments (upper). Live imaging of Cy5-UTP (magenta) and UTR-Rps4x-Venus fusion or UTR-Venus (green) reporter before and 0, 5, and 10 min after photobleaching of the Venus (green) fluorescence. The yellow arrowheads indicate the sites of co-localization, and the white lines indicate the outlines of axons. Scale bar, 2 μm. (E) Plot showing Pearson’s correlation coefficient of pixel intensities between Cy5-UTP signals and recovered Venus signals (Venus-Rps4x [n = 8] or Venus-only [n = 12]). Bars, average with SEM, Mann-Whitney *U* test, ^∗^p ≤ 0.05.

To investigate the interaction of axonally synthesized RPs with the ribosome, we first performed an *in situ* proximity ligation assay (PLA), a technology capable of detecting the close physical association between 2 molecules (<400 Å) (Yoon et al., 2012). We carried out metabolic labeling of newly synthesized proteins with l-azidohomoalanine (AHA) in severed axons (Dieterich et al., 2007). After biotin conjugation to AHA via “click” chemistry, we performed PLA to detect the association between axonally synthesized proteins (biotin antibody) and ribosomes, using the anti-rRNA antibody Y10B (Aakalu et al., 2001) (Figure 4A). As eukaryotic ribosomes are 250–300 Å in diameter, the proximity between the AHA-labeled RPs and the rRNA is expected to generate a PLA signal if axonally synthesized RPs are incorporated into axonal ribosomes (Figure 4A). To eliminate the detection of elongating nascent AHA-labeled polypeptide chains, axons were treated with 200 μM puromycin, which releases growing polypeptide chains from the ribosome (Cioni et al., 2019, Nathans, 1964) before the sample fixation. We observed a clear PLA signal in the axons, which was abolished by anisomycin treatment (Figures 4B and 4C). We also found that the PLA signal was significantly higher in Netrin-1-stimulated axons than in unstimulated axons (Figures 4B and 4C), which is consistent with the results that Netrin-1 promotes axonal RP synthesis (Figures 2 and S2). While these proximity-based results do not rule out the association of newly synthesized non-RP factors with the surface of axonal ribosomes, they demonstrate that some proteins synthesized in response to Netrin-1 in axons become closely associated on-site with ribosomes and support the idea that newly synthesized RPs join axonal ribosomes.

To look specifically at the question of whether newly synthesized RPs themselves co-localize with ribosomes, we used a modified FRAP approach and dual-channel time-lapse imaging in live axons. We have previously shown that blastomere-injected labeled UTP is incorporated predominantly into ribosomal RNAs (Wong et al., 2017) and that translation takes place on these Cy5-UTP granules in live retinal axons (Wong et al., 2017, Cioni et al., 2019). Therefore, we used Cy5-UTP as a live reporter of ribosome/RNA-rich granules. We also introduced a cDNA encoding Venus-Rps4x/eS4, an RP fusion protein. We observed that bright fluorescent puncta of Venus-Rps4x/eS4, but not the Venus-only control, co-localize and co-move with Cy5 fluorescent puncta, confirming that Cy5-UTP labels ribosome/RNA-rich granules (Figures 4D and S4). Next, to visualize the newly synthesized Venus proteins, we performed photobleaching of the Venus fluorescence with 488-nm laser light and then examined the co-localization between Cy5-UTP fluorescence and the recovered Venus signal. Before photobleaching, we cut the axons to exclude the signal of Venus proteins transported from the soma. Immediately post-cut and before photobleaching, we added Netrin-1 to stimulate axonal Rps4x/eS4 synthesis. Time-lapse imaging revealed that the signal of recovered (axonally synthesized) Venus-Rps4x/eS4 protein, but not of control Venus protein, exhibited sustained co-localization with Cy5-labeled RNA after photobleaching in severed somaless RGC axons (Figures 4D and S4). This result was further confirmed by a spatial correlation analysis, in which the correlation (Pearson’s correlation coefficient) of the pixel intensities between recovered Venus-Rps4x and Cy5-UTP signals was significantly higher than that between Venus-only control and Cy5-UTP (Figure 4E).

**Figure 5:**
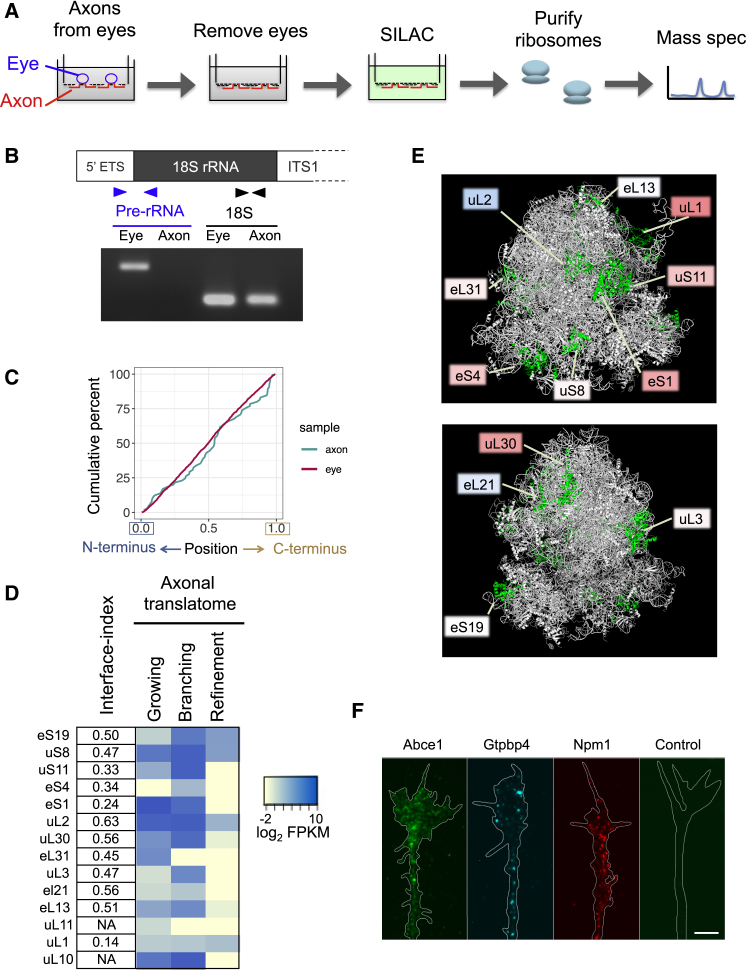
Axonally Synthesized RPs Physically Interact with the Ribosome in a Nucleolus-Independent Manner (A) Experimental strategy of axon purification, SILAC labeling, and axonal ribosome purification. (B) Diagram and gel image of RT-PCR detection of mature 18S rRNA and pre-rRNA. (C) Cumulative percentage of the relative position of labeled peptides detected in axonal (cyan) and eye (red) ribosome samples. (D) List of RPs whose labeled peptides were detected in the axonal ribosomes. The heatmap shows the translation levels of RPs in 3 different developmental stages of *in vivo* mouse RGC axons. (E) Human 80S ribosome structure with indication of the RPs whose labeled peptides were detected in the axonal ribosomes. The colors of the squares represent the interface-index of each labeled RP (see Figure 3). (F) Immunostaining of ribosomal assembly factors and a secondary antibody-only control in cultured *Xenopus laevis* RGC axons. Scale bar, 5 μm.

To obtain biochemical evidence for the axonal incorporation of axonally synthesized RPs into axonal ribosomes, we combined axon-pSILAC (Cagnetta et al., 2018) with axonal ribosome purification (Figure 5A). In this method, we labeled newly synthesized proteins with heavy amino acids for 3 h in somaless axons cultured in a Boyden chamber. Together with heavy amino acids, Netrin-1 was added to stimulate the axonal translation of RPs. After the lysis of labeled axons, axonal ribosomes were purified using a sucrose cushion and ultracentrifugation, followed by the identification of newly synthesized proteins with mass spectrometry (Figure 5A). To obtain sufficient axonal material, ∼2,000 eyes were cultured for each sample. Mature 18S rRNA was detected in both somas and axons, whereas the pre-rRNA, which is present only in the nucleolus, was detected only in somata (Figure 5B), confirming that there was no contamination of the axon samples with somata/nuclei in these experiments. We used the same method as a control sample on isolated eyes that were treated with heavy amino acids for 48 h. Mass spectrometry analysis revealed that ∼93% of all detected proteins in the axonal ribosome samples were in a single gene-network cluster that includes RPs and ribosome-interacting proteins, validating the ribosome purification procedure (Figure S5A). Mass spectrometry detected a number of labeled (i.e., axonally synthesized) RP peptides in the axonal ribosome samples. The ratios of labeled/unlabeled RP peptides in the axonal ribosome samples were highly variable among RPs, whereas relatively constant ratios of labeled RPs were observed in whole cells (the eye sample) (Figure S5B). These results not only show that locally synthesized RPs physically associate with ribosomes in the axon but they also exclude the possibility that the detection of labeled RPs in the axonal ribosome samples is caused by contamination of our axon sample with cell bodies. We still detected labeled RP peptides in axons even when the axon lysate was treated with RNase A/T1 and puromycin, which dissociate mRNA-binding proteins, including the poly(A)-binding protein from the purified ribosomes (Figures S5C–S5F). This result suggests that the detection of these labeled RP peptides was not due to the binding of free extra-ribosomal RPs to the ribosome-bound mRNAs. The puromycin step also ensured that we were not detecting growing polypeptide chains, which is consistent with the fact that the position of heavy labeled RP peptides was not biased toward the N terminus (Figure 5C). Analysis of our mouse *in vivo* axonal translatome showed that most (85%) of the labeled RPs detected in the axonal ribosome samples are axonally synthesized during the branching stage of RGC axon development (Figure 5D). Most (91%) of these are not deeply penetrated RPs (Figure 3B) in the ribosome structure (2.2-fold depletion of interface-index < 0.6) (Figures 5D and 5E). Although the subcellular nature of our material prohibited the use of approaches to resolve whether the axonally synthesized RPs are actually embedded in the ribosome in their normal positions, these results support the idea that locally synthesized RPs physically associate with axonal ribosomes in a nucleolus-independent manner.

To explore the possibility that nucleolar ribosome assembly factors mediate the axonal interaction between RPs and ribosomes (Kressler et al., 2010), we searched our *Xenopus* RGC axon proteome data (Cagnetta et al., 2018) for proteins whose mouse ortholog genes were annotated with the GO terms “nucleolus” (GO: 0005730) or “ribosome assembly” (GO: 0042255). We found that 12.6% (124 of 982) of proteins with these GO terms were detected in our axon sample, some of which were confirmed by immunostaining (Figure 5F). Many of these factors were detected in the axons of different types of neurons, such as mouse callosal axons (Poulopoulos et al., 2019) and axons of rat cortical neurons (Chuang et al., 2018), suggesting that the functions of these factors are conserved among these neuron types (Figure S5G).

### Locally Synthesized Rps4x/eS4 Is Required to Maintain Ribosome Function in Axons

**Figure 6:**
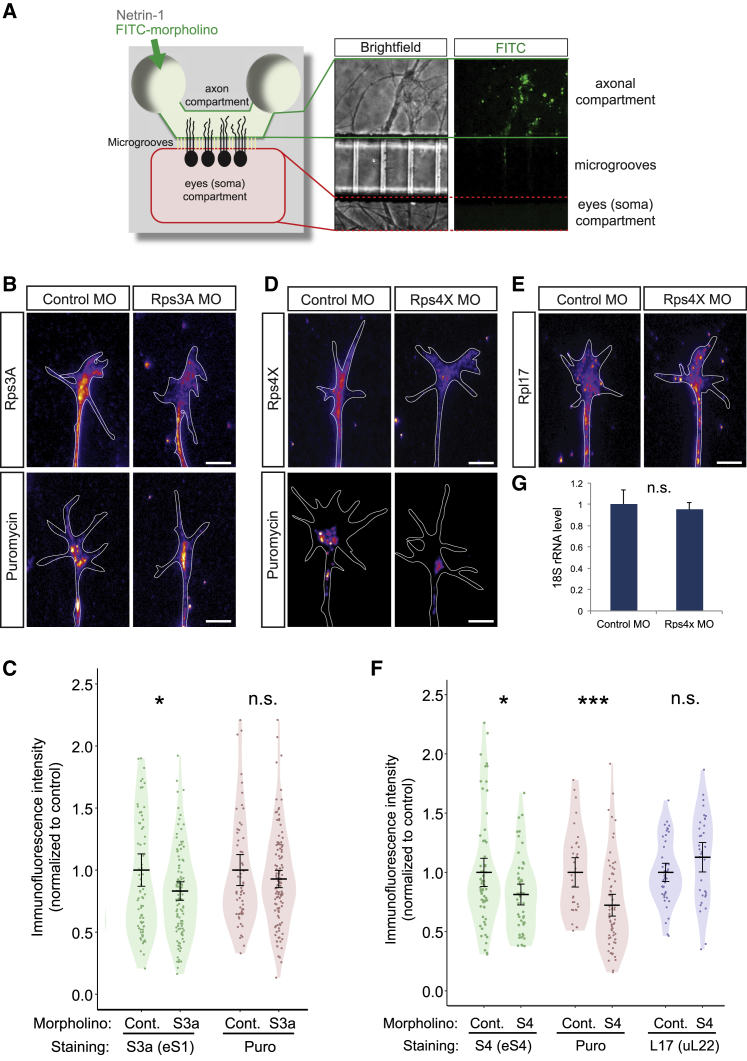
Locally Synthesized Rps4x/eS4 Is Required to Maintain Ribosome Function in Axons (A) Diagram (left) and image of RGC axons after FITC-morpholino introduction in a microfluidic chamber. (B–F) Images (B, D, and E) and QIF plots (bars, average, 95% CI, and distribution of normalized levels) (C and F) of Rps3a/eS1, Rps4x/eS4, Rpl17/uL22, and puromycin immunostaining in axons treated with control morpholinos (Cont.) or with morpholinos against *rps3a* (eS1) (B and C) or against *rps4x* (eS4) (D–F), with Welch t test (^∗^p = 0.030, C; 0.015, F; ^∗∗∗^p = 0.0004; n.s., not significant). (G) qRT-PCR quantification, normalized to control MO, of 18S rRNA in axonal samples treated with control or *rps4x* (eS4) morpholinos (n.s., not significant in Mann-Whitney *U* test).

Next, we focused on the functional interaction between axonally synthesized RPs and axonal ribosomes. To ask whether local RP synthesis affects the protein synthesis activity of axonal ribosomes, we performed axon-specific inhibition of RP synthesis using a microfluidic chamber that isolates the axonal compartment from the cell body compartment (Taylor et al., 2005). Taking advantage of the fluidic isolation of the chamber (Taylor et al., 2005), we delivered a FITC-conjugated morpholino only into the axonal compartment to inhibit axonal RP synthesis (Figure 6A) for three RPs—Rps3a/eS1, Rps4x/eS4, and Rpl5/uL18—all of which are synthesized/translated robustly in RGC axons (Cagnetta et al., 2018, Shigeoka et al., 2016). Although we could not find a significant effect on axonal protein synthesis after *rps3a* (eS1) or *rpl5* (uL18) morpholino delivery, we found that the *rps4x* (eS4) morpholino significantly reduced the level of proteins labeled with puromycin, a structural analog of aminoacylated-tRNA which labels newly synthesized proteins (Starck et al., 2004, tom Dieck et al., 2015) in axonal growth cones (Figures 6B–6D, 6F, S6C, and S6D). The *rps4x* morpholino decreases the Rps4x/eS4 protein level only in the axons but not in the somata (Figures 6D, 6F, Figures S6A and S6B). In addition, we found that neither the level of Rpl17/uL22 nor the level of 18S rRNA was changed by the *rps4x* morpholino (Figures 6E–6G), suggesting that the decreased level of newly synthesized proteins in axons is not caused by a reduced number of axonal ribosomes. These data show that the inhibition of axonal *rps4x/eS4* translation decreases the level of translation activity in the axon, suggesting a crucial role for axonally synthesized Rps4x/eS4 in axonal ribosome function.

### Axonal Synthesis of Rps4x/eS4 Is Crucial for Axon Branching *In Vivo*

**Figure 7:**
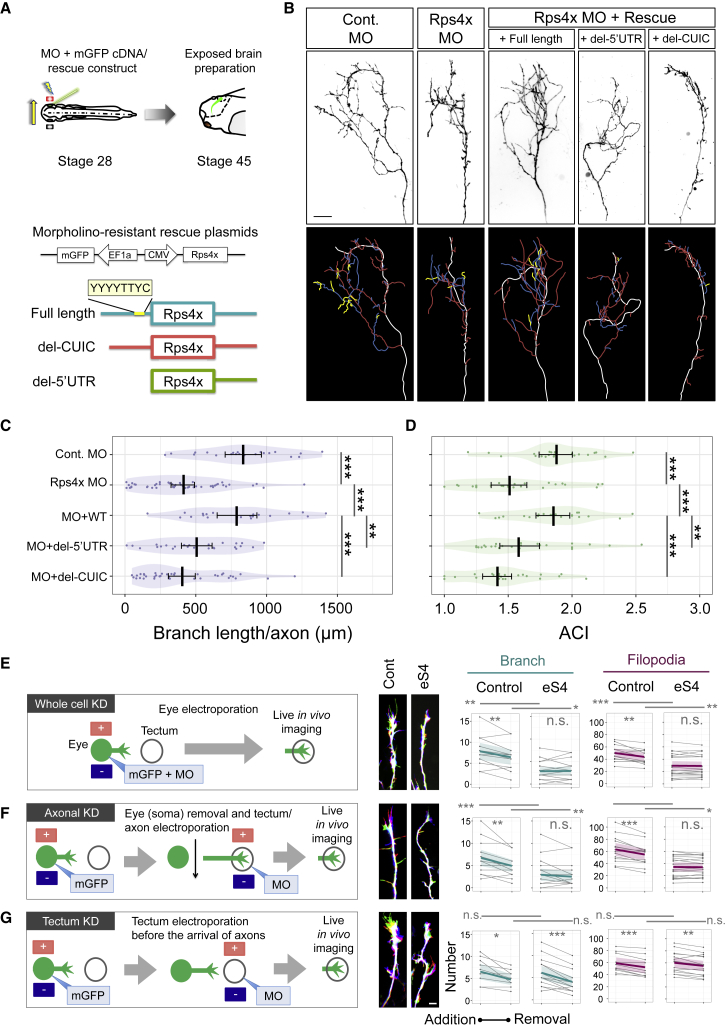
Axonal Translation of Rps4x/eS4 Is Crucial for Axon Branching *In Vivo* (A) Experimental workflow (upper) for eye electroporation and live image acquisition of axon branches/arbors. Dual promoter constructs (lower) used for rescue conditions. (B) Lateral view of a single *in vivo* RGC axon in the tectum with color-coded images of axon shaft (white), primary (red), secondary (blue), and tertiary (yellow) branches. (C and D) Average, 95% CI, and distribution of total branch length per axon (C) and axon complexity index (ACI; see Figure S7B for formula) (D) in the embryos electroporated with the morpholino/rescue constructs (1-way ANOVA with 2-stage step-up method of Benjamini, Krieger, and Yekutiei multiple comparisons test); n = 21 (Cont. MO), 47 (MO), 21 (MO + WT), 25 (MO + del-5′ UTR), and 37 (MO + del-CUIC). (E–G) Experimental workflow for each knockdown (KD) experiment (left) and quantification (right) of *in vivo* axon branching in control MO^−^ and rps4x MO^+^ axons after eye electroporation (whole-cell KD) (n = 12, Cont.; 20, Rps4x) (E) and tectum electroporation at stages 41–43 (axonal KD) (middle, n = 14, Cont.; 19, Rps4x) (F), and at stages 35–38 (tectum KD) (lower, n = 10, Cont.; 15, Rps4x) (G). Images (middle panel) show a merged overlay of 3 time points (0, 5, and 10 min in blue, red, and green, respectively). Line graphs (right) show the number of added/removed branches/filopodia (paired and unpaired t test, ^∗∗^p < 0.01, ^∗∗∗^p < 0.001; n.s., not significant).

Next, we asked whether newly synthesized RPs incorporated into axonal ribosomes are critical for axon development *in vivo*. Our analysis showed that the axonal synthesis of RPs is enhanced by Netrin-1, which promotes axon branching (Dent et al., 2004, Manitt et al., 2009), and it peaks at the branching stage in mouse RGC axons *in vivo* (Figure 1B). A previous study demonstrated that exposed intact brains treated acutely with protein synthesis inhibitors show a reduction in axonal branching dynamics in the optic tectum (Wong et al., 2017). Thus, our finding that Rps4x/eS4 knockdown inhibits axonal protein synthesis (Figure 6) prompted us to test whether the local synthesis of Rps4x/eS4 is essential for the normal branching of axons. Taking advantage of an *in vivo* system to visualize single *Xenopus* RGC axons (Wong and Holt, 2018, Wong et al., 2017), we inhibited Rps4x/eS4 synthesis by morpholino-based knockdown (Figure 7A). *In vivo* electroporation of *rps4x* morpholino into stage 28 eyes significantly reduced the number, length, and complexity of RGC axon branches in the optic tectum at stage 45 (Figures 7A–7D and S7A–S7C). These branching phenotypes were rescued by a morpholino-resistant *rps4x* cDNA with full-length UTRs, but not by an *rps4x* cDNA lacking either the entire 5′ UTR or the CUIC motif, suggesting that the CUIC-mediated regulation of Rps4x/eS4 synthesis is crucial for axon branching (Figures 7B–7D and S7A–S7C). We next investigated the role of Rps4x/eS4 synthesis in the dynamics of axon branching. We first performed *in vivo* live imaging of axons after morpholino electroporation into the eye at stage 28 (Wong et al., 2017). In control embryos at stages 41–43, we observed a significant bias in the addition over the removal of filopodia and branches, in agreement with previous work showing that this bias helps to build arbor complexity (Wong et al., 2017). Inhibition of Rps4x/eS4 synthesis in RGCs abolished this bias. In addition, the numbers of filopodia and branches being added and removed were both reduced after *rps4x* knockdown (Figures 7E and S7D).

To test whether these phenotypes were caused by the inhibition of local axonal rather than somal translation of *rps4x*, we delivered the *rps4x* morpholino directly into arborizing RGC axons in the tectum by electroporation at stages 41–43 (Figure 7F) (Yoon et al., 2012). To eliminate the possibility that the intracellular diffusion of the morpholino affects somal ribosome biogenesis and the number of ribosomes in the RGC axon, we physically removed the eye, which contains the RGC cell bodies, before morpholino delivery (Figure 7F) (Wong et al., 2017). Similar to the global inhibition of *rps4x* translation in RGCs, local inhibition of axonal *rps4x* translation abolished the addition/removal bias and reduced the number in both filopodia and branches (Figures 7F and S7E). To evaluate the extent of secondary effects due to morpholino-electroporated tectal cells, we delivered the morpholino locally into the tectum before the arrival of RGC axons (stages 35/36–37/38) and subsequently visualized the branching dynamics of axons after tectal entry at stages 41–43. No significant differences were observed in branching dynamics, indicating that the inhibition of Rps4x/eS4 synthesis in tectal cells is not responsible for the observed branching defects in RGC axons (Figures 7G and S7F). These results indicate that the CUIC-mediated axonal synthesis of Rps4x/eS4 is critical for the proper arbor development of RGC axons *in vivo* (Figure S7G).

## Discussion

Our results provide multiple independent lines of evidence that are consistent with the hypothesis that locally synthesized RPs in RGC axons are incorporated into axonal ribosomes and are required to maintain axonal ribosome function and normal neuronal developmental processes. Since the distal regions of axons are far away from their somata, our findings suggest the possibility that they have adopted a nucleolus-independent mechanism for the on-site repair and/or modification of their ribosomes through the local supply of newly synthesized RPs. Since we could not detect 18S pre-rRNA in axons (Figure 5B), it is unlikely that *de novo* assembly of ribosomes takes place in axons. A more plausible hypothesis is that free, extra-ribosomal RPs in axons are incorporated into pre-existing ribosomes in the axon through the replacement or addition of components. This model is consistent with recent proteomics and cryoelectron microscopy studies, which showed the presence of ribosomes lacking specific RP components in eukaryotic cells (Shi et al., 2017, Slavov et al., 2015, Samir et al., 2018). It seems possible that axonal RP synthesis plays an important role in ribosome maintenance, particularly for the surface ribosomal components that may be prone to damage (Figures 3 and S3). It is also possible, however, that it modifies the ribosomal composition to generate “specialized’’ ribosomes (Genuth and Barna, 2018, Kondrashov et al., 2011, Shi et al., 2017, Xue et al., 2015), which are tuned to preferentially translate specific mRNAs that regulate axon function.

We found that free extra-ribosomal RPs are enriched in axons compared to the whole cell (Figure S3). This may partly explain the much higher frequencies of the ribosomal incorporation of newly synthesized RPs in Netrin-1-stimulated axons (Figure S5B) than those predicted from previous turnover studies performed on whole cells (Dice and Schimke, 1972, Lastick and McConkey, 1976). As Netrin-1-induced axonal RP synthesis may significantly increase the concentration of free RPs in axons, it may also elicit a transient increase in ribosome incorporation of newly synthesized RPs in axons (Figure 5). An important question related to this is whether the ribosomal incorporation of RPs in axons needs any catalytic activity of *trans*-acting auxiliary factors. In prokaryotes, a factor-free *in vitro* assembly of functional ribosomal subunits has been successfully demonstrated by bringing together purified rRNAs and RPs (Pulk et al., 2010, Sykes and Williamson, 2009), but this has not yet been reported in eukaryotes. Accumulating evidence suggests that a large number of non-ribosomal factors (>200) and small nucleolar RNAs are involved in the *de novo* ribosome assembly in eukaryotic cells, but most of these factors are related to rRNA processing or transport of RPs/pre-ribosomes, while only a few factors have been demonstrated to support the incorporation of RPs (Kressler et al., 2010). We found that surface components of the ribosomal subunits are enriched in the axons. The binding of RPs to the surface of the ribosome may therefore be a simpler and different process compared to *de novo* ribosome biogenesis, which comprises the processing and folding of the pre-rRNA and its sequential assembly with deeply embedded ribosomal proteins. It is also possible that some ribosome assembly factors have roles in the axonal incorporation of some RPs. We detected a considerable number of ribosome assembly factors in the axon, although we have not identified any axonal function for these factors. To fully understand the mechanisms underlying axonal RP incorporation, further work on the axonally detected ribosome assembly factors is needed.

We identified a motif called CUIC, which is a form of pyrimidine-rich element (PRE) in the region immediately upstream of the initiation codon of many axonally translated RP mRNAs. It is known that the 5′ TOP sequence plays an important role in the translational control of mRNAs, including RP-coding mRNAs, downstream of the mTOR signaling pathway (Levy et al., 1991). We showed that alternative short isoforms of RP-coding transcripts truncated at the CUIC region are highly enriched in the axon, suggesting the possibility that CUIC can function as a 5′ TOP-like sequence for these short isoforms in axons. This finding may provide an integrated explanation for functions of the 5′ TOP motif and other previously reported pyrimidine-rich sequences, such as the pyrimidine-rich translational element (PRTE) (Hsieh et al., 2012). Our analysis using previous CLIP-seq studies revealed that, in addition to the eIF3 components, TIA1 and TIAL1, proteins involved with N6-methyladenosine (m6A) methylation (RBM15 and RBM15B) and the recognition of m6A (YTHDF1, YTHDF2, and YTHDC1) (Patil et al., 2016) are specifically associated with the CUIC region (Figure 1E). Since a recent study reported that an m6A in the 5′ UTR directly binds eIF3, which is sufficient to recruit the 43S complex to initiate translation in a cap-independent manner (Meyer et al., 2015), RNA methylation at regions surrounding the CUIC motif core sequence and the subsequent recruitment of the eIF3-43S complex may be involved in the CUIC-mediated axonal translation. Since CUIC is located immediately upstream of the initiation codon, one interesting possibility is that CUIC changes the distance between the 5′ end and the initiation codon through alternative 5′ end formation, affecting the positional relation among initiation factors and ribosomes on the mRNA, which in turn may affect the speed or reliability of translation in response to a signal.

In this study, we uncovered crucial functions of locally synthesized Rps4x/eS4 in axonal mRNA translation and in proper axon branching *in vivo*. Although we cannot exclude the possibility that some unknown extra-ribosomal function of Rps4x/eS4 is responsible for the axon branching phenotype, we propose that the intra-ribosomal function of Rps4x/eS4 is more likely to explain the phenotype observed after axonal inhibition of Rps4x/eS4 synthesis, since several lines of evidence suggest that the process of axon branching is highly dependent on local translation. A previous study showed that RNA granules dock at sites of branch emergence and invade stabilized branches and that acute inhibition of axonal translation by protein synthesis inhibitors and downregulation of the eIF2α pathway cause a very similar axon branching defect as observed after Rps4x/eS4 knockdown (Cagnetta et al., 2019, Wong et al., 2017). Furthermore, our previous analysis of the developmental changes of the axonal translatome (Shigeoka et al., 2016) showed that the number of axonally translated mRNAs is highest during branching stages, when synapses are being formed. A recent proteomic study showed that Rps4x/eS4 is the most significantly sub-stoichiometric among all of the RPs in polysomal ribosomes of eukaryotic cells (Slavov et al., 2015), suggesting an interesting possibility that, compared to Rps4x/eS4-containing ribosomes, Rps4x/eS4-deficient ribosomes have less translation elongation activity, which may cause a polysomal “traffic jam” on the mRNA (Chu et al., 2014). These results suggest the possibility that axonal RP translation induced by Netrin-1 and the CUIC motif may make axonal ribosomes competent for the extensive protein synthesis needed for axon arborization and synaptogenesis (Figure S7G). Collectively, our findings suggest that ribosomes may be dynamic structures in axons, exchanging/repairing components in response to extrinsic signals. Further studies are needed to provide a fuller understanding of how RP mRNA translation in axons contributes to fundamental processes that establish and/or maintain neural circuits.

## STAR★Methods

### Key Resources Table

**Table:** 

REAGENT or RESOURCE	SOURCE	IDENTIFIER
**Antibodies**		

Rabbit polyclonal anti-Rps4x	Proteintech	Cat#14799-1-AP; RRID: AB_2238567
Rabbit polyclonal anti-Rpl17	Proteintech	Cat#14121-1-AP; RRID: AB_2253985
Rabbit polyconal anti-Rps14	Abcam	Cat#ab174661
Mouse monoclonal anti-Rpl19	Abcam	Cat#ab58328; RRID: AB_945305
Mouse monoclonal anti-Rps23	Abcam	Cat#ab57644; RRID: AB_945314
Rabbit polyclonal anti-Rps3a	Proteintech	Cat#14123-1-AP; RRID: AB_2253921
Mouse monoclonal anti-Neurofilament A	DSHB	Cat#3A10; RRID: AB_531874
Rabbit polyclonal anti-Rpl39	Abcam	Cat#ab74740; RRID: AB_1524345)
Rabbit polyclonal anti-Rps12	Proteintech	Cat#16490-1-AP; RRID: AB_2146233
Rabbit monoclonal anti-Gtpbp4	Abcam	Cat#ab92342; RRID: AB_2049721
Mouse monoclonal anti-Npm1	Origene	Cat#BM5524; RRID: AB_1008764
Rabbit polyclonal anti-Abce1	Abcam	Cat#ab32270; RRID: AB_722514
Rabbit polyclonal anti-β-actin	Abcam	Cat#ab8227; RRID: AB_2305186
Rabbit polyclonal anti-β-catenin	Sigma-Aldrich	Cat#C2206; RRID: AB_476831
Mouse monoclonal anti-puromycin, clone 12D10	Sigma-Aldrich	Cat#MABE343; RRID: AB_2566826
Mouse monoclonal anti-ribosomal RNA (Y10B)	Abcam	Cat#ab171119
Rabbit monoclonal anti-biotin	Cell Signaling	Cat#5597; RRID: AB_10828011
Goat anti-rabbit Alexa Fluor 568	Abcam	Cat#ab150117; RRID: AB_2688012
Goat anti-mouse Alexa Fluor 594	Abcam	Cat#ab150080; RRID: AB_2650602
Mouse monoclonal anti-puromycin, clone 12D10, Alexa Fluor 647 Conjugate	Sigma-Aldrich	Cat#MABE343-AF647
Goat anti-mouse HRP	Abcam	Cat#ab6789; RRID: AB_955439
Goat anti-rabbit HRP	Abcam	Cat#ab97080; RRID: AB_10679808

**Bacterial and Virus Strains**		

BioBlue Chemically Competent Cells	Bioline	Cat#BIO-85037

**Chemicals, Peptides, and Recombinant Proteins**		

AHA (L-Azidohomoalanine)	ThermoFisher	Cat#C10102
Leibovitz’s L-15 Medium	ThermoFisher	Cat#11415064
Leibovitz’s L-15 medium -Lys -Arg	GIBCO Life technologies	N/A (customized)
Stable isotope-coded amino acids Lys8	Silantes GmbH	Cat#211603902
Stable isotope-coded amino acids Arg10	Silantes GmbH	Cat#20160390
Antibiotic-Antimycotic (100X)	Thermo Fisher Scientific	Cat#15240062
Poly-L-lysine	Sigma-Aldrich	Cat#P1274
Laminin	Sigma-Aldrich	Cat#L2020
Puromycin	Sigma-Aldrich	Cat#P8833
Cycloheximide	Sigma-Aldrich	Cat#C4859
Anisomycin	Sigma-Aldrich	Cat#A9789
Cy5-UTP	PerkinElmer	Cat#NEL582001EA
SUPERase In RNase Inhibitor	Ambion	Cat#AM2696
FluorSave	Merck Millipore (Calbiochem)	Cat#345789-20
Duolink In Situ Mounting Medium with DAPI	Sigma-Aldrich	Cat#DUO82040-5ML
Recombinant mouse Netrin-1	R&D systems	Cat#1109-N1
RNase A	Ambion	Cat#EN0531
Rnase T1	Ambion	Cat#EN0541
n-Octylglucoside	Sigma-Aldrich	Cat#10634425001

**Critical Commercial Assays**		

NeuroPORTER transfection reagent	Sigma-Aldrich	Cat#NPT01
Rneasy mini kit	QIAGEN	Cat#74104
SuperScript II First Strand Synthesis kit	Thermo Fisher Scientific	Cat#18080051
Quantitect SYBR Green PCR kit	QIAGEN	Cat#204141
KAPA HyperPrep kit	Roche	Cat#KK8503
NextSeq 500/550 high output v2 kit (150 cycles)	Illumina	Cat#FC-404-2002
Duolink *In Situ* Detection Reagents Green	Sigma-Aldrich	Cat#DUO92014
Duolink *In Situ* PLA probe Anti-Rabbit PLUS	Sigma-Aldrich	Cat#DUO92002
Duolink *In Situ* PLA probe Anti-Mouse MINUS	Sigma-Aldrich	Cat#DUO92004
Click-iT Cell Reaction Buffer Kit	Thermo Fisher Scientific	Cat#10269

**Deposited Data**		

Proteomics data	This study	PRIDE: PXD011032
		PRIDE: PXD015574
RNA-sequencing data	This study	GSE135502

**Experimental Models: Organisms/Strains**		

*Xenopus laevis*	Nasco	Cat#LM00715, Cat#LM00535

**Oligonucleotides**		

Morpholino: Control MO 5′-CCTCTTACCTCAGTTA CAATTTATA-3′	Gene tools	N/A
Morpholino: Rps4x.S MO 5′-TTCTTCGGTCCGCGA GCCATG-3′	Gene tools	N/A
Morpholino: Rps4x.L MO 5′-CTTTTTCGGTCCACG AGCCATTTTC-3′	Gene tools	N/A
Morpholino: Rpl5 MO 5′- ACCTTTACGAACCCC ATTTTGCTCT −3′	Gene tools	N/A
Morpholino: Rps3a.S MO 5′- TCTTGTTCTTGCCG ACTGCCATC −3′	Gene tools	N/A
Morpholino: Rps3a.S MO 5′- GTTCTTGCCCACTGC CATCTTGC −3′	Gene tools	N/A
Primers for 18S rRNA (*Xenopus laevis*) qRT-PCR forward: 5′-GTAACCCGCTGAACCCCGTT-3′	This study	N/A
Primers for 18S rRNA (*Xenopus laevis*) qRT-PCR reverse: 5′-CCATCCAATCGGTAGTAGCG-3′	This study	N/A
5′RACE oligo 1: 5′-GGTCCACGAGCAAAGACA CCAGTCAA −3′	This study	N/A
5′RACE oligo 1^st^ PCR: 5′- GCAAAGACACCAGTCA ACTTGTCCAACATC −3′	This study	N/A
5′RACE oligo 2^nd^ PCR: 5′- AACACGCTTCAAGTGCT TTTTCGGTCCA −3′	This study	N/A
5′RACE sequencing oligo: 5′- GTGCTTTTTCGGT CCACGAGCAAAGAC −3′	This study	N/A
Primers for 18S pre-rRNA (*Xenopus laevis*) RT-PCR forward: 5′-GAGCGAGAGAGAAAGACGGA −3′	This study	N/A
Primers for 18S pre-rRNA (*Xenopus laevis*) RT-PCR reverse: 5′-TCTAGAGTCACCAAAGCGGC −3′	This study	N/A

**Recombinant DNA**		

Plasmid: pCS2+-Venus-Rps4x-Full length	This study	N/A
Plasmid: pCS2+-Venus-Rps4x-Del CUIC	This study	N/A
Plasmid: pCS2+-Venus-Rps4x-Del 5′UTR	This study	N/A
Plasmid: pCS2+-Venus-Rps4x-Del 5′UTR-3′UTR	This study	N/A
Plasmid: pCS2+-Venus-Rps4x (Xenopus)	This study	N/A
Plasmid: pCS2+-mGFP	Das et al., 2003	N/A
Plasmid: pCS2+-mRFP	Poggi et al., 2005	N/A
Plasmid: pCS2+-Mo resistant Rps4x del-5’UTR/mGFP dual promotor	This study	N/A
Plasmid: pCS2+-Mo resistant Rps4x del-CUIC/mGFP dual promotor construct	This study	N/A
Plasmid: pCS2+-MO resistant Rps4x/mGFP dual promotor construct	This study	N/A

**Software and Algorithms**		

Volocity	PerkinElmer	Version 6.0.1, RRID:SCR_002668
R	v.3.2.2	RRID: SCR_001905
TopHat2	v.2.0.12	RRID: SCR_013035
Cufflinks	v.2.2.1	RRID: SCR_014597
HISAT2	v.2.1.0	RRID: SCR_015530
HOMER	v.3.0	RRID: SCR_010881
Vienna RNA	v.2.4.3	RRID: SCR_008550
DAVID	v.6.8	RRID: SCR_001881
FIJI	Schindelin et al., 2012	Version 2.0.0-rc-65/1.51w, RRID:SCR_002285
Simple Neurite Tracer	Longair et al., 2011	https://imagej.net/Simple_Neurite_Tracer
MATLAB	Mathworks; v. R2016b	RRID:SCR_001622
GraphPad PRISM	Graphpad; v.5	RRID:SCR_002798
MaxQuant	v.1.4.1.2	RRID: SCR_014485
RapidSTORM	Wolter et al., 2012	N/A
PyMOL	https://www.pymol.org2/2	RRID:SCR_000305

**Other**		

RNA-seq dataset	Shigeoka et al., 2016	GEO: GSE79352
RNA-seq dataset	Ding et al., 2017	GEO: GSM1948717
Proteome dataset	Cagnetta et al., 2018	PRIDE: PXD005469
POSTAR2 database	Zhu et al., 2019	N/A
Microfluidic chambers	Xona Microfluidics	Cat#SOC150
Falcon Cell Culture Inserts	Thermo Fisher Scientific	Cat#08-771-7/353102

### Lead Contact and Materials Availability

Further information and request for resources and reagents should be directed to the Lead Contact, Christine E. Holt (ceh33@cam.ac.uk). All unique plasmids are available from the Lead Contact.

### Experimental Model and Subject Details

#### *Xenopus laevis* Embryos

*Xenopus laevis* eggs were fertilized *in vitro* and embryos were raised in 0.1x Modified Barth’s Saline (MBS; 8.8mM NaCl, 0.1 mM KCl, 0.24mM NaHCO_3_, 0.1 mM HEPES, 82μM MgSO_4_, 33μM Ca(NO_3_)_2_, 41μM CaCl_2_) at 14-20°C and staged according to the tables of Nieuwkoop and Faber (1994). All animal experiments were approved by the University of Cambridge Ethical Review Committee in compliance with the University of Cambridge Animal Welfare Policy. This research has been regulated under the Animals (Scientific Procedures) Act 1986 Amendment Regulations 2012 following ethical review by the University of Cambridge Animal Welfare and Ethical Review Body (AWERB).

#### Primary *Xenopus* Retinal Cultures

Eye primordia were dissected from Tricaine Methanesulfonate (MS222) (Sigma-Aldrich) anesthetized embryos of either sex at stage 35/36 and cultured on 10μg/ml poly-L-lysine (Sigma-Aldrich)- and 10μg/ml laminin (Sigma-Aldrich)-coated glass bottom dishes (MatTek) in 60% L-15 medium (ThermoFisher), 1x Antibiotic-Antimycotic (ThermoFisher) at 20°C for 24-48 hours. 10-20 eye primordia (from 5-10 embryos) were cultured per dish and, typically, 2-3 dishes were used per experimental condition for each biological replicate. Replicates in each experiment using *Xenopus laevis* in this study were obtained from different batches of embryos.

### Method Details

#### RNA-seq analysis of *Xenopus laevis* axons

We performed the axon cultures using eyes of stage 33/34-37/38 embryos of *Xenopus laevis* (Nieuwkoop and Faber, 1994) on a Boyden chamber device as described above at room temperature for 48hrs. To obtain the axonal transcriptome, we isolated total RNA from RGC axons separated from their cell bodies by a Boyden chamber device (1 μm pore, Falcon Cell Culture Inserts, 10289270/353102, Thermo fisher scientific) coated on both sides of the membrane with poly-L-lysine (10 μg/ml) and only on the bottom side with laminin (10 μg/ml). We cultured 500 eyes of *Xenopus laevis* embryos (stage 33/34-37/38) for each sample, yielding < 4-5μg axonal material. Eyes were dissected out and cultured as whole eyes on the upper surface of the transfilter in 60% L15 medium containing penicillin streptomycin fungizone (GIBCO) at room temperature for 48 hours. After 48 hours, we removed the cell bodies and lysed the axons in RLT buffer (QIAGEN) containing β-mercaptoethanol. RNA was then extracted using the RNeasy Mini kit (QIAGEN) followed by in-column DNase I treatment to remove genomic DNA contamination. We then amplified cDNA using a method developed for single cell transcriptomics (Tang et al., 2009) with minor modifications (Shigeoka et al., 2016). The cDNA library preparation was performed using a KAPA Hyperprep kit (Roche) and cDNA libraries were subjected to a RNA-sequencing run on Next-seq 500 instrument (Illumina) using the 150 cycles high output kit (Illumina). The sequence reads were mapped using HISAT 2 version 2.1.0, and FPKM values were estimated using Cufflinks version 2.1.1. If RP-coding mRNAs are transcribed from two homeologs, we calculated the average FPKM of homeologs. We used previously published RNA-seq data (GSM1948717) of *Xenopus laevis* whole embryos for a control.

#### Plasmid Construction

To construct Venus reporter plasmids used in FRAP and single molecule translation imaging, Venus cDNA and 5′ / 3′ UTR of mouse Rps4x/eS4 (NM_009094) were integrated into BamHI-XbaI sites of pCS2+ (University of Michigan, Ann Arbor, Mich.). 8 nucleotides (CTCTTTCC) in the 5′ UTR were deleted in the “Del-motif” construct. To generate Rps4x-Venus fusion constructs, Venus and *X.laevis* Rps4x.S sequences were inserted into the BamHI-RcoRI sites of pCS2+. In these constructs, a CMV promoter drives the expression of 5′ UTR(Rps4x.S)-Venus-linker (Gly-Gly-Ser-Gly-Gly-Gly-Ser-Gly)-CDS (Rps4x.S, NM_001097003.1)-3′ UTR(Rps4x.S). Because frog 5′ UTR sequences of Rps4x.S in public databases could be truncated, we used a sequence of actually transcribed mRNA in frog embryos, which is obtained from a previously published RNA-seq data (GSM1948717) mapped to genome Xla.v91: 5′-cgcgctctcttcctgccagagttcagcgcgcactctttatcccggcgggaccggaaggaggaggtcttttcc-3′. To construct the plasmids for the rescue experiment of the morpholino phenotype, we replaced the β-actin cDNA of the morpholino insensitive β-actin/mGFP dual promoter construct (Wong et al., 2017) with 5′ UTR-CDS-3′ UTR of Rps4x.S in which silent mutations (ATGGCTCGCGGACCGAAGAAGC = > ATGGCACGGGGCCCCAAAAAAC) were introduced to avoid morpholino binding. In the “del-CUIC” rescue construct, both of the two motifs (TCTCTTCC and TCTTTTCC) in the 5′ UTR were removed.

#### Axon Culture and SILAC

We performed the axon cultures as described above for RNA-seq analysis. Then, we treated the eyes with lysine- and arginine-free L15 (60%) medium for 1hr. After eye removal to eliminate the cell bodies, the axons were cultured in L15 depletion medium containing “heavy” amino acids (84 μg/ml [13C6,15N4] l-arginine, 146 μg/ml [13C6,15N2] l-lysine (Silantes, Germany) and Netrin-1 (600 ng/ml; R&D systems) for 3hrs. Soma removal was confirmed by absence of nuclear DAPI staining. For the preparation of control eye samples, we cultured dissected eyes in L15 depletion medium containing “heavy” amino acids at room temperature for 48hrs. Lysis of axons was performed using 500 μL Lysis buffer (9mM Tris-HCl pH 7.4, 270mM KCl, 9 mM MgCl_2_, 1% n-octylglycoside (Sigma-Aldrich),100μg/ml cycloheximide (Sigma-Aldrich), 0.5mM DTT, EDTA-free protease inhibitor cocktail (Roche) and SUPERase In RNase Inhibitor (Ambion)). Lysates were centrifuged at 16.000 g at 4°C for 15 min and the supernatant was transferred to an ice-cold 1.5ml tube. For the puromycin/RNaseA/T1 treated sample, axons were treated with 200 μM puromycin for 15min before lysis and lysates were treated with 10 μg/μl RNase A (Ambion) and 250U RNase T1 (Ambion) for 15 min at 25°C.

#### Polysome Fractionation

For density gradient fractionation, the lysate was layered on a sucrose gradient (10%–50%) and ultracentrifugation was performed using a Beckman SW-40Ti rotor and Beckman Optima L-100 XP ultracentrifuge, with a speed of 35,000 rpm at 4°C for 160 min. Fractionations and UV absorbance profiling were carried out using Density Gradient Fractionation System (Teledyne ISCO). For sucrose cushioning, the lysate was layered on a 20% sucrose solution (20% sucrose, 10 mM Tris-HCl pH 7.4, 300 mM KCl,10 mM MgCl2), which contains a high concentration of KCl to avoid the aspecific binding of proteins to ribosomes. Then, ultracentrifugation was performed using a Beckman SW-55Ti rotor and Beckman Optima L-100 XP ultracentrifuge, with a speed of 41,000rpm at 4°C for 120 min. Proteins were precipitated from each fraction using methanol-chloroform precipitation and pellets were resuspended in 1x NuPAGE LDS sample buffer and used for western blotting as described below. RNA from each fraction was isolated as described below.

#### Quantitative PCR

RNA from fractionated samples or from the axonal compartment of the microfluidic chambers were isolated using the RNeasy mini kit (QIAGEN) and reverse transcribed into cDNA using random hexamers and the SuperScript III First-strand synthesis kit (Thermo Fisher Scientific). Triplicate reactions for qPCR were prepared using this cDNA and the Quantitect SYBR Green PCR kit (QIAGEN) according to manufacturer’s instructions. Plates were centrifuged shortly and run on a LightCycler 480 machine (Roche) using the following PCR conditions: denaturation step for 15 s at 94°C; annealing step for 30 s at 60°C; extension step for 30 s at 72°C. The following primers were used for qPCR: *18S rRNA* 5′-GTAACCCGCTGAACCCCGTT-3′ and 5′-CCATCCAATCGGTAGTAGCG-3′.

#### 5′RACE and RT-PCR

We used 5′ RACE System for Rapid Amplification of cDNA Ends, version 2.0 (Thermo Fisher scientific) and followed. For the cDNA synthesis, we used the primer: 5′-GGTCCACGAGCAAAGACACCAGTCAA-3′. For the 1st PCR amplification, we used a reverse primer: 5′-GCAAAGACACCAGTCAACTTGTCCAACATC-3′. For the 2nd PCR amplification, we used a reverse primer: 5′-AACACGCTTCAAGTGCTTTTTCGGTCCA-3′.

We purified the amplified product from gel after the electrophoresis by using Wizard SV Gel and PCR Clean-Up System (Promega) and performed the direct sequence of the purified DNA using the primer: 5′-GTGCTTTTTCGGTCCACGAGCAAAGAC-3′. For the amplification of pre-rRNA, the following primers were used: *18S-5end-F* 5′-GAGCGAGAGAGAAAGACGGA-3′ and *18S-5end-R* 5′- TCTAGAGTCACCAAAGCGGC-3′.

PCR amplifications were performed using Ex Taq HS (Takara).

#### Axonal Morpholino Delivery *in vitro*

Modified microfluidic chambers (Xona microfluidics, SOC150) were pre-coated with poly-L-lysine (10 μg/ml) and laminin (10 μg/ml). Eyes dissected from stage 30-33 *X. laevis* embryos were plated in the open chamber of SOC150. RGC axons were grown in 60% L15 medium containing μg/ul penicillin streptomycin fungizone at room temperature for 48 hr. For the morpholino introduction, we prepared two solutions: diluted transfection reagent (2 μL NeuroPORTER Transfection Reagent (Sigma-Aldrich) with 5.5 μL L15 (60%)) and morpholino solution (2.5 μL of 1mM morpholino oligonucleotide (mixture of 5′-CTTTTTCGGTCCACGAGCCATTTTC-3′ (against Rps4x.L) and 5′-TTCTTCGGTCCGCGAGCCATG-3′ (against Rps4x.S) or 5′-ACCTTTACGAACCCCATTTTGCTCT-3′ (against Rpl5) or a mixture of 5′-TCTTGTTCTTGCCGACTGCCATC-3′ (against Rps3a.S) and 5′-GTTCTTGCCCACTGCCATCTTGC-3′ (against Rps3a.L) with 5 μL L15 (60%)). We mixed 7.5 μL morpholino solution with 7.5 μL diluted transfection reagent and incubated the mixture at room temperature for 5 min. We added the 15 μL of mixture directly to 200 μL of the medium present in the axon chamber and incubated it at room temperature for 18-24 hours.

#### Immunohistochemistry

Microfluidic chamber cultures were treated with 600ng/ml Netrin-1 (R&D systems) for 20 minutes and then fixed after detaching microfluidic chambers from the glass bottom dishes in 2% formaldehyde/7,5% sucrose in PBS for 20 min at 20°C. The fixed cultures were steamed for 10min in sodium citrate buffer for antigen retrieval in case of ribosomal protein staining. Subsequently, they were permeabilized for 3-5 min in 0.1% Triton X-100 in PBS and blocked with 5% heat-inactivated goat serum in PBS for 45 min at 20°C. Primary antibodies were incubated overnight at 4°C, followed by Alexa Fluor-conjugated secondary antibodies for 45 min at 20°C in the dark. Cultures were mounted in FluorSave (Calbiochem).

Antibodies were used at the following dilutions. Primary antibodies: rabbit anti-Rps4x (Proteintech, Cat#14799-1-AP; RRID: AB_2238567, 1:200), 1:200 rabbit anti-Rpl17 (Proteintech, Cat#14121-1-AP; RRID: AB_2253985, 1:200), rabbit-anti-Rps3a (Proteintech, Cat#14123-1-AP; RRID: AB_2253921). Secondary antibodies: goat anti-rabbit Alexa Fluor 594 (Abcam, Cat#ab150080; RRID: AB_2650602, 1:1000). Culture medium in the axonal compartment was replaced with 200 μL of culture medium containing 600ng/ml Netrin-1 and 10μg/ml puromycin (Sigma). After 20min, the axonal compartment was washed once with fresh culture medium before detaching the microfluidic chamber from the dish. The retinal culture was immediately fixed in 2% formaldehyde/7,5% sucrose in PBS for 20 min at 20°C, permeabilized for 3-5 min in 0.1% Triton X-100 in PBS, blocked with 5% heat-inactivated goat serum in PBS for 30 min at 20°C and then labeled with Alexa Fluor 647-conjugated mouse anti-puromycin antibody (Millipore, Cat#MABE343-AF647, 1:250) overnight at 4°C. Cultures were mounted in FluorSave (Calbiochem).

Transfilters from Boyden chambers were immunostained after eye removal using mouse-anti-neurofilament A (Developmental Studies Hybridoma Bank, Cat#3A10; RRID: AB_531874) and DAPI, as previously described (Cagnetta et al., 2018).

For qIF or immunostaining on regular *Xenopus* retinal cultures (Figures 2D, 2E, 5F, S2D, and S2E), cultures were treated with or without 50 μM cycloheximide (Sigma) and 600ng/ml Netrin-1 for 5 minutes before fixation in 2% formaldehyde/7,5% sucrose in PBS for 20 min at 20°C. For Rps12 qIF, 600ng/ml Netrin-1 was added for 30 minutes before fixation. The fixed cultures were steamed for 10min in sodium citrate buffer for antigen retrieval in case of ribosomal protein and Npm1 staining. For Abce1 and Gtpbp4 immunostaining, methanol fixation was used. The cultures were then permeabilized and blocked as described above and stained with primary antibodies were incubated overnight at 4°C, followed by Alexa Fluor-conjugated secondary antibodies for 45 min at 20°C in the dark. Cultures were mounted in FluorSave (Calbiochem). The following antibodies were used: rabbit anti-Rps14 (Abcam, Cat#ab174661; 1:200), rabbit anti-Rps4x (Proteintech, Cat#14799-1-AP; RRID: AB_2238567, 1:200), rabbit anti-Rpl39 (Abcam, Cat#ab74740; RRID: AB_1524345, 1:200), rabbit anti-Rps12 (Proteintech, Cat#16490-1-AP; RRID: AB_2146233, 1:200), rabbit anti-Abce1 (Abcam, Cat#ab32270; RRID: AB_722514, 1:200), rabbit anti-Gtpbp4 (Abcam, Cat#ab92342; RRID: AB_2049721, 1:200), mouse anti-Npm1 (Origene, Cat#BM5524; RRID: AB_1008764, 1:200), Secondary antibodies: goat anti-rabbit Alexa Fluor 594 (Abcam, Cat#ab150080; RRID: AB_2650602, 1:1000), goat anti-mouse Alexa Fluor 568 (Abcam, Cat#ab150117; RRID: AB_2688012, 1:1000).

Randomly selected non-collapsed growth cones were imaged at 60x on a Nikon Eclipse TE2000-U inverted microscope equipped with an EMCCD camera. For Abce1, Gtpbp4 and Npm1 immunostaining, imaging was carried out at 100x on a Perkin Elmer Spinning Disk UltraVIEW ERS, Olympus IX81 inverted microscope. Exposure time was kept constant and below gray-scale pixel saturation.

#### Blastomere Microinjection

Embryos were microinjected with Cy5-UTP at 100 μM in a total volume of 5nl (PerkinElmer) into both of the dorsal blastomeres at 4- or 8-cell stage (Wong et al., 2017). Embryos were first de-jellied in 2% Cysteine (Sigma-Aldrich) in 1x MBS (pH 8.0), washed 3 times in 0.1x MBS and aligned on a grid in 4% Ficoll (Sigma-Aldrich) in 0.1x MBS with 1X antibiotic-antimyotic (Thermo Fisher Scientific). Injections were performed using glass capillary needles (outer diameter: 1.0mm; inner diameter: 0.5mm; Harvard Apparatus) and a pressurized microinjector (Picospritzer, General Valve).

#### FUNCAT-rRNA Proximity Ligation Assay

*Xenopus* retinal explants from stage 35-36 embryos were first cultured as described above in complete 60% L-15 medium (GIBCO, Thermo Fisher Scientific) for 24 hours, which was then replaced with methionine-free L-15 medium (GIBCO, Thermo Fisher Scientific) for a further 12 hours. After 11 hours, 200μM anisomycin (Sigma-Aldrich) was added into the culture for the +anisomycin condition and an equal concentration and volume of DMSO (Sigma-Aldrich) was added to all other conditions. After 1 hour, axons were severed from the explants to exclude axonal transport of nascent peptides synthesized in the cell bodies, immediately followed by the addition of 1mM AHA (Thermo Fisher Scientific) and 600ng/ml Netrin-1 (R&D) in the +AHA +Netrin-1 and +anisomycin conditions, 1mM AHA in the +AHA -Netrin condition, or an equal concentration and volume of DMSO (as in the AHA stock solution) and 600ng/ml Netrin-1 in the -AHA condition. After 45 minutes, 200μM puromycin (Sigma-Aldrich) was mixed into the AHA- and/or Netrin-1-containing medium for a further 15 min incubation to release all synthesizing polypeptides from axonal ribosomes. After washing once with PBS to remove unincorporated AHA, the cultures were fixed in 2% formaldehyde/7.5% sucrose in PBS for 20 min at 20°C and permeabilized for 5 min in 0.1% Triton X-100 in PBS. The Click Chemistry reaction was performed according to the manufacturer’s protocol (Click-iT Cell Reaction Buffer Kit, Thermo Fisher Scientific) starting with washing the fixed cultures with 2% BSA in PBS (w/v) and incubating the culture for 30 min at 20°C in freshly prepared Click-iT reaction cocktail containing the Click-iT cell reaction buffer, the buffer additive, CuSO_4_ and 5μM biotin-alkyne (Thermo Fisher Scientific). The cultures were then washed once in 2% BSA in PBS (w/v) and blocked with 5% heat-inactivated goat serum in PBS for 30 min at 20°C. A mouse anti-ribosomal RNA (Y10B) antibody (1:100, Abcam, Cat#ab171119) and a rabbit anti-biotin antibody (1:200, Cell Signaling, Cat#5597, RRID: AB_10828011) were incubated overnight at 4°C. The proximity ligation assay was carried out according to the manufacturer’s protocol (Duolink, Sigma-Aldrich) with minor modifications. Dishes were washed twice for 5 minutes with 0.002% Triton X-100 in PBS and incubated with anti-rabbit (+) and anti-mouse (-) PLA probes for 1 hour at 37°C, with ligase for 30 min at 37°C and with the polymerase mix with green fluorescence for 100 min at 37°C. The samples were subsequently mounted with mounting medium (Duolink, Sigma-Aldrich) and imaged using an Olympus IX81 inverted microscope fitted with a PerkinElmer Spinning Disk UltraVIEW VoX using a 60x silicone oil objective (1.4 N.A., Olympus), and an ORCA-Flash4.0 V2 CMOS camera (Hamamatsu). Volocity 6.3.0 software (PerkinElmer) was used for acquisition. The number of discrete fluorescent puncta within a randomly chosen 50μm axon segment in each image was counted using Volocity software.

#### Fluorescence Recovery After Photobleaching

Retinal cultures for FRAP assays were obtained from eyes of stage 33/34 embryos expressing one of the four Venus constructs (Figures 2F and S2F) or Venus-Rps4x / Venus-only for Cy5-UTP colocalization experiments (Figures 4D, 4E, and S4). These constructs were introduced by eye-targeted electroporation at stage 26 as described in previous studies (Falk et al., 2007, Wong et al., 2017). 1μg/μl Venus construct (Figures 2F and S2F) or 1.5μg/μl of Venus-Rps4x (Venus only) with 100uM Cy5-UTP (Figures 4D, 4E, and S4) were injected for the electroporation.

FRAP imaging was performed on an Olympus IX81 inverted microscope equipped with a PerkinElmer Spinning Disk UltraVIEW VoX and a 60x (NA, 1.30) Olympus silicone oil immersion objective. Images were acquired with an ORCA-Flash4.0 V2 CMOS camera (Hamamatsu) using Volocity software (PerkinElmer). Photobleaching was performed using an UltraVIEW PhotoKinesis device (PerkinElmer). Photobleaching was performed at 85%–90% laser power (488 nm laser line) with 20–30 bleach cycles.

For the FRAP experiment imaging axons expressing Rps4x UTR-containing Venus constructs or the Venus-only construct indicated in Figures 2F and S2F, axons of the 24h retinal cultures were severed from the eye and 600ng/ml Netrin-1 was added into the culture. A randomly selected fluorescent growth cone of a severed axon then proceeded to the photobleaching step using 488 nm laser line, immediately after pre-photobleaching fluorescent and phase contrast images were acquired. The photobleached area was manually defined so that growth cones and > 50 μm of the axon shaft were bleached (thus reducing likelihood of fluorescence recovery resulting from Venus diffusion from unbleached areas of the axon shaft). In anisomycin-treated condition, 24h cultures were incubated with 100μM anisomycin for 20 min before severing the axons. Time-lapse post-photobleaching images were captured at 1 min intervals using a 488 nm laser line at 20% laser power, together with phase contrast images for the corresponding time point. Exposure time was adjusted to avoid pixel saturation.

For the FRAP experiment imaging axons expressing Cy5-UTP and Venus-Rps4x fusion construct or Venus-only construct shown in Figures 4D and S4, axons of the 24h retinal cultures were severed from the eye and 600ng/ml Netrin-1 was added into the culture. A 30 s pre-photobleaching movie at maximum speed of a randomly selected Cy5- and Venus-positive 50 μm axon segment was taken, followed by photobleaching of the Venus fluorescence using a 488 nm laser line. 30 s post-photobleaching fluorescent and phase contrast movies at maximum speed were acquired at 0 min, 5 min and 10 min after the completion of photobleaching. Exposure time was adjusted to avoid pixel saturation.

#### Single Molecule Translation Imaging

Single molecule translation imaging was done as previously described(Ströhl et al., 2017, Tatavarty et al., 2012). Embryos at stage 26 were electroporated with plasmids expressing Rps4x UTR-containing Venus constructs or the Venus-only and left in 0.1X MBS to continue to develop. Venus-expressing eyes from electroporated embryos at stage 34 were dissected and cultured. After 24 hours, a non-collapsed fluorescent growth cone was randomly selected and, prior to the bleaching step, imaged with low irradiance (< 2W/cm^2^) in both fluorescence and bright field mode to generate an outline image. The growth cone was then photobleached for 10-30 s with an irradiance of 1.5 kW/cm2 to eliminate the fluorescence. A reduced laser intensity of 0.3 kW/cm2 was used to ensure survival of the axons while simultaneously bleaching newly synthesized Venus proteins. The flash-like recovery of Venus fluorescence recorded with an exposure time of 200 ms for 180 s. After that another bright field image was taken to check for survival. Retracted growth cones were excluded from analysis. In Netrin-1-stimulated conditions, 600ng/ml of Netrin-1 was bath applied immediately before the photobleaching step. All imaging steps were performed under epifluorescence illumination. An EM gain of 200 was used on the EMCCD camera to ensure single molecule sensitivity. The field of illumination was twice the size of the imaged field of view to bleach diffusing or transported fluorescent proteins from outside the growth cone before entering the field of view. Imaging was performed on a custom-made inverted single-molecule fluorescence microscope built around a commercial microscope frame (Olympus IX73). The illumination laser wavelength was at 488nm (Coherent Sapphire) for excitation of the YFP derivate Venus in combination with a 525/645-emission filter (Semrock) and a dichroic beam splitter (Chroma ZT405/488/561/640rpc). The laser beam was circularly polarized to excite fluorescent proteins homogeneously regardless of their orientation. The microscope was equipped with an EM-CCD camera (Andor iXon Ultra 897) with effective pixel size on the sample of 118 nm. A 100x NA = 1.49 oil immersion TIRF objective (Olympus) was used.

#### *In Vivo* Knockdown and Imaging

Targeted eye and tectal electroporations were performed as previously described (Falk et al., 2007, Wong et al., 2017). Stage 28 embryos were anesthetized in 0.4mg/ml MS222 in 1X MBS. The retinal primordium was injected with electroporation mixture, followed by electric pulses of 50ms duration at 1000ms intervals, delivered at 18V (please refer to the list below for the mixture and the number of electric pulses delivered for each experiment). The embryos were recovered and raised in 0.1X MBS until the desired embryonic stage for experiment.

1) Mature axon visualization (Figures 7B–7D and S7): 1μg/μl of pCS2+mGFP (or 1μg/μl of pCS2+mGFP/MO resistant Rps4x rescue dual promoter construct cDNA for rescue experiments), 0.5mM control/Rps4x MO; 1 pulse.
2) Axon branching dynamics (Figures 7E–7G and S7): 1μg/μl of pCS2+mGFP or 1μg/μl of pCS2+mRFP, 0.5mM control/Rps4x MO; 4 pulses.

For tectal electroporation (Figures 7 and S7), the lateral surface of the hemisphere of the brain contralateral to the eye labeled with mRFP (electroporated at Stage 28 as described above) was exposed by careful removal of overlying eye and epidermis. 8X 18V electric pulses of 50ms duration at 1000ms intervals were delivered immediately after the 1mM control/Rps4x MO was locally ejected at the vicinity of the target area. The procedure was repeated once to ensure efficient delivery of the MO (Wong et al., 2017). Embryos were lightly anaesthetized with 0.4mg/ml MS222 in 1xMBS. The lateral surface of the brain contralateral to the electroporated eye was exposed by carefully removing the overlying epidermis and the contralateral eye. The electroporated eyes were also surgically removed to prevent somal contribution of proteins in Figures 7F and S7. Embryos were mounted in an oxygenated chamber created with Permanox slides (Sigma-Aldrich) and Gene Frame (ThermoFisher) and bathed in 1xMBS with 0.1mg/ml MS222, for visualization with fluorescence microscopy. Imaging was performed using 40X (NA 1.25) or 60X UPLSAPO objectives (NA 1.3) with a PerkinElmer Spinning Disk UltraVIEW ERS, Olympus IX81 inverted spinning disk confocal microscope. Z stack intervals of 1-2μm were employed for acquiring images with Volocity (PerkinElmer).

#### Western Blotting

Proteins were resolved by SDS-PAGE on NuPage 4%–12% Bis-Tris gels (Invitrogen) and transferred to a nitrocellulose membrane (Bio-Rad). The blots were blocked in blocking buffer (5% milk in TBS-T) and then incubated with primary antibodies in blocking buffer overnight at 4°C. After 3 washes (5 minutes each) with TBS-T, the blots were incubated with HRP-conjugated secondary antibodies in blocking buffer for 1 hour at RT, washed again for 3 times (5 minutes each) in TBS-T and developed using ECL-based detection (Pierce ECL plus, Thermo Scientific). The following primary antibodies were used for western blot analysis: mouse anti-Rpl19 (Abcam, Cat#ab58328; RRID: AB_945305, 1:1000), mouse anti-Rps23 (Abcam, Cat#ab57644; RRID: AB_945314, 1:1000), rabbit anti-Rps4X (Proteintech, Cat#14799-1-AP; RRID: AB_2238567, 1:1000), rabbit β-catenin (Sigma-Aldrich, Cat#C2206; RRID: AB_476831, 1:8000) and rabbit anti-β-actin (Abcam, Cat#ab8227; RRID: AB_2305186).

#### Mass Spectrometry

1D gel bands were transferred into a 96-well PCR plate. The bands were cut into 1mm^2^ pieces, de-stained, reduced (DTT) and alkylated (iodoacetamide) and subjected to enzymatic digestion with chymotrypsin overnight at 37°C. After digestion, the supernatant was pipetted into a sample vial and loaded onto an autosampler for automated LC-MS/MS analysis. LC-MS/MS experiments were performed using a Dionex Ultimate 3000 RSLC nanoUPLC (Thermo Fisher Scientific Inc., Waltham, MA, USA) system and a Q Exactive Orbitrap mass spectrometer (Thermo Fisher Scientific Inc, Waltham, MA, USA). Separation of peptides was performed by reverse-phase chromatography at a flow rate of 300nL/min and a Thermo Scientific reverse-phase nano Easy-spray column (Thermo Scientific PepMap C18, 2 μm particle size, 100A pore size, 75 μm i.d. x 50cm length). Peptides were loaded onto a pre-column (Thermo Scientific PepMap 100 C18, 5 μm particle size, 100A pore size, 300 μm i.d. x 5mm length) from the Ultimate 3000 autosampler with 0.1% formic acid for 3 minutes at a flow rate of 10 μL/min. After this period, the column valve was switched to allow elution of peptides from the pre-column onto the analytical column. Solvent A was water + 0.1% formic acid and solvent B was 80% acetonitrile, 20% water + 0.1% formic acid. The linear gradient employed was 2%–40% B in 30 minutes. The LC eluant was sprayed into the mass spectrometer by means of an Easy-Spray source (Thermo Fisher Scientific Inc.). All m/z values of eluting ions were measured in an Orbitrap mass analyzer, set at a resolution of 70000 and was scanned between m/z 380-1500. Data-dependent scans (Top 20) were employed to automatically isolate and generate fragment ions by higher energy collisional dissociation (HCD, NCE:25%) in the HCD collision cell and measurement of the resulting fragment ions was performed in the Orbitrap analyzer, set at a resolution of 17500. Singly charged ions and ions with unassigned charge states were excluded from being selected for MS/MS and a dynamic exclusion window of 20 s was employed. For the label-free quantification of proteins, peptide identification and relative quantification was carried out in Proteome Discoverer version 2.3. A standard label free quantification workflow was utilized with the Mascot search algorithm, against a *Xenopus laevis* proteins downloaded from Xenbase. The search parameters included: trypsin as the proteolytic enzyme, with maximum of two missed cleavages; variable oxidation modification of methionine, and deamidation of asparagine and glutamine; fixed carbamidomethylation modification of cysteine; precursor and fragment mass tolerances of 20 ppm and 0.1 Da respectively. The false discovery rate (FDR) was set at < 1% with two peptide matches to proteins considered as reliable. For the analysis of SILAC labeled proteins, we used Maxquant in addition to Proteome Discoverer since it is the most commonly used software for SILAC analysis. Raw data were processed using Maxquant (version 1.6.1.0) (Cox and Mann, 2008) with default settings. MS/MS spectra were searched against the *X. laevis* protein sequences from Xenbase (xlaevisProtein.fasta). Enzyme specificity was set to trypsin/P, allowing a maximum of two missed cleavages. The minimal peptide length allowed was set to seven amino acids. Global false discovery rates for peptide and protein identification were set to 1%. The match-between runs and re-quantify options were enabled. To avoid false positives, we analyzed only those RPs where more than two labeled peptides were detected by the software or if the MS spectrum of the detected labeled peptides showed clear peaks at the expected m/z value.

### Quantification and Statistical Analysis

#### Statistics

The n number for each experiment, details of statistical analysis and software are described in the figure legends or main text. Statistical analyses used in this study include one-way ANOVA, Welch’s t test, Mann-Whitney U test, Kolmogorov–Smirnov test and Fisher’s exact Test. Statistical significance is defined as, n.s., not significant, ^∗^p < 0.05, ^∗∗^p < 0.01, ^∗∗∗^p < 0.001. Statistical analysis was performed using R version 3.2.2 or Prism (GraphPad).

#### Bioinformatics Analysis

We analyzed the developmental change of level of all mRNAs translated in the mouse RGC axons in dataset (GSE79352). The sequence reads were mapped to the mouse genome (mm10) using TopHat 2 version 2.0.12 with default settings, except for the “–read- realign- edit-dist 0” option. Transcript assembly and estimation of FPKM (Fragments Per Kilobase of transcript per Million fragments sequenced) values were performed using Cufflinks version 2.2.1. For RNA-seq analysis of frog RGC axons, the sequence reads were mapped to the *X. laevis* v9.2 genome (Xenbase) using HISAT2 2.1.0 with default settings. Transcript assembly and estimation of FPKM (Fragments Per Kilobase of transcript per Million fragments sequenced) values were performed using Cufflinks version 2.1.1. For the GO enrichment analysis, we only analyzed genes showing FPKM > 1 in both of two replicates. For analysis of the pSILAC-SP3 results, we extracted all proteins that show a significant change (FDR < 0.01) by stimulation (5min or 30min) of Netrin-1. We performed a GO enrichment analysis using DAVID 6.8 with default settings for BP-direct, MF-direct and CC-direct categories and used all detected proteins as the background of the enrichment calculation. For the ribosome structure analysis, we used the PyMOL function InterfaceResidues (https://pymolwiki.org/index.php/Main_Page) to know the interface residues of RPs with rRNAs. We analyzed the structure of human 80S ribosome (6ek0) published previously (Natchiar et al., 2017). For the small ribosomal subunit proteins, we analyzed interface residues between RPs and 18S rRNA. For the large ribosomal subunit proteins, we analyzed the interface residues of RPs with 5S, 5.8S and 28S rRNAs.

#### Motif Analysis

All sequences of mouse cDNAs were retrieved from BioMart at Ensembl (GRCm38, ensemble Genes 91). *De novo* motif analysis was performed using HOMER version 3.0 with custom FASTA files containing all 5′ UTR sequences of mouse RP-coding cDNAs. For the analysis of CUIC-containing genes, we selected all genes whose mRNA contains the 8-mer nucleotides (T/C)(T/C)(T/C)(T/C)TT(T/C)C located less than 100nt upstream of the initiation codon. The secondary structure analysis of UTRs was performed using performed using RNAfold in the ViennaRNA package version 2.4.3 with default settings. The conservation among species of all CUIC-containing RP mRNAs was calculated from phastCons60way.UCSC.mm10. For the analysis of proteins binding the CUIC region, we used Human RBP binding sites data downloaded from the POSTAR2 database (Zhu et al., 2019). To visualize the binding sites of EIF components, we created custom tracks on the UCSC genome browser.

#### Quantification of Immunofluorescence

For quantitation of fluorescence intensity, the growth cone outline was traced on the phase contrast image using Volocity version 6.0.1 (PerkinElmer), then superimposed on the fluorescent image. The software calculated the fluorescent intensity within the growth cone, giving a measurement of pixel intensity per unit area. The growth cone outline was then placed in an adjacent area clear of cellular material to record the background fluorescent intensity. This reading was subtracted from the growth cone reading, yielding the background-corrected intensity.

#### FRAP analysis

Quantification of fluorescence intensity was performed using Volocity software (PerkinElmer). At each time point, the outline of the growth cone was traced using phase contrast images. Mean gray values from the 488-channel were subsequently calculated as mean pixel intensity per unit area within the specified region of interest (ROI). This ROI was then placed in an adjacent area clear of cellular material to record the background fluorescent intensity. This reading was subtracted from the growth cone reading, yielding the background-corrected intensity. Unhealthy axons exhibiting signs of photo-toxicity after FRAP (characterized by blebbing, growth cone collapse and/or retraction) were excluded from analysis. In addition, only growth cones of axons extending more than 100 μm from the eye explant were quantified to reduce effects of somal diffusion. Relative fluorescent recovery (R) at each time point was calculated by the formula: Rx = (Ix – Ipost) / (Ipre – Ipost). Where, Ix = normalized fluorescent intensity of the growth cone ROI at time point ‘x’, Ipre = normalized fluorescent intensity before photobleaching and Ipost = normalized fluorescent intensity immediately after photobleaching (t = 0mins). Significance was tested using a two-way ANOVA. Colocalization analysis between Cy5-RNA granules and Venus-Rps4x (N = 8 axons) or Venus (N = 12 axons) in Figure 4E was performed on the first image of a 30 s movie taken 10min post-photobleaching. In each image, a 5um axon segment containing Cy5-RNA granules and recovered Venus-Rps4x or Venus signal was chosen and the Pearson’s correlation coefficient between Cy5 and Venus channels within the selected area was measured by Volocity software.

#### Single Molecule Translation Imaging Analysis

Translation event counting was performed by manual counting supplemented with the previously reported software-assisted automated event detection(Ströhl et al., 2017), where localizations of individual protein translation events were retrieved using maximum likelihood estimation with a Gaussian model fit via the software package rapidSTORM. A threshold of ∼6700 ADC, corresponding to ∼500 photons per localization, was applied to filter out noise and non-Venus blinking events. This threshold was found by manual selection of Venus flashes and determination of the “average” photon budget of a single emitting Venus molecule. The tracking option of rapidSTORM was used to recombine photons emanating from the same Venus protein over multiple frames. All events in a small area around the growth cones were included for analysis due to the high mobility of filopodia. Results are normalized to the growth cone area, thus given as event/s/μm^2^.

#### Branching Analysis

A filopodium was defined as a protrusion with length < 5 μm while a branch was defined as a protrusion with length > 5 μm (Drinjakovic et al., 2010, Hörnberg et al., 2013, Kalous et al., 2014, Wong et al., 2017). Data were analyzed in PRISM 7 (GraphPad). ‘n’ represents the number of axons. ^∗^p < 0.05, ^∗∗^p < 0.01, ^∗∗∗^p < 0.001, #p < 0.05, ##p < 0.01, ###p < 0.001. Details of statistic results such as p values, degree of freedom, and t/F values are presented in the figure legends. For axon arbor analysis, 3D projection of axon arbors acquired at 40X were semi-automatically traced through the z axis using the Simple Neurite Tracer plugin (Longair et al., 2011) in Fiji. The resulting traces were then analyzed for the number and the length of axon branches as well as the Axon Complexity Index (ACI) (Marshak et al., 2007). These measured parameters were compared using one-way ANOVA with the Two-stage step-up method of Benjamini, Krieger and Yekutieli multiple comparisons test. Cumulative distribution curves of total branch number represent least-squares fits to a Gaussian function and were compared using Extra sum-of-squares F test. The proportions of simple (ACI < 1.4) and complex (ACI ≥ 1.4) arbors in different groups were compared using Fisher’s exact test (Drinjakovic et al., 2010). For analysis of branching dynamics, the numbers of protrusions added and removed were counted on the terminal 50 μm of mGFP/mRFP-labeled RGC axons for 10 min (imaged at an interval of 30 s) (Wong et al., 2017). The addition and removal of protrusions were then compared statistically. A paired t test was used for intragroup comparison and unpaired t test was used for intergroup comparisons.

#### Western Blot Analysis

Developed films from western blot detection were scanned and imported into FIJI. The color was inverted and the background corrected signals for Rps4x and β-catenin were measured. Measured Rps4x levels were then normalized to β-catenin to obtain a ratiometric readout. A paired t test was used to assess differences in Rps4X protein levels between control MO and Rps4X MO samples (n = 3 independent experiments).

### Data and Software Availability

The accession numbers for the mass spectrometry proteomics data reported in this paper are PRIDE: PXD011032 and PRIDE: PXD015574. The accession number for the RNA-seq data reported in this paper is GEO: GSE135502.

## Supplemental Information

Document S1. Figures S1–S7

Table S1. CUIC Genes, Related to Figure 1

Table S2. Regulated Protein Synthesis, Related to Figure 2

Table S3. Enriched GOs, Related to Figure 2

Document S2. Article plus Supplemental Information
