# Supplementary material for: On-Site Ribosome Remodeling by Locally Synthesized Ribosomal Proteins in Axons
Source: Cell Rep. 2019 Dec 10;29(11):3605–3619.e10. doi: 10.1016/j.celrep.2019.11.025 (PMC6915326; doi:10.1016/j.celrep.2019.11.025)
Supplement: Document S1. Figures S1–S7 [file mmc1.pdf]

Cell Reports, Volume *29*

Supplemental Information

On-Site Ribosome Remodeling by Locally Synthesized Ribosomal Proteins in Axons

Toshiaki Shigeoka, Max Koppers, Hovy Ho-Wai Wong, Julie Qiaojin Lin, Roberta Cagnetta, Asha Dwivedy, Janaina de Freitas Nascimento, Francesca W. van Tartwijk, Florian Ströhl, Jean-Michel Cioni, Julia Schaeffer, Mark Carrington, Clemens F. Kaminski, Hosung Jung, William A. Harris, and Christine E. Holt

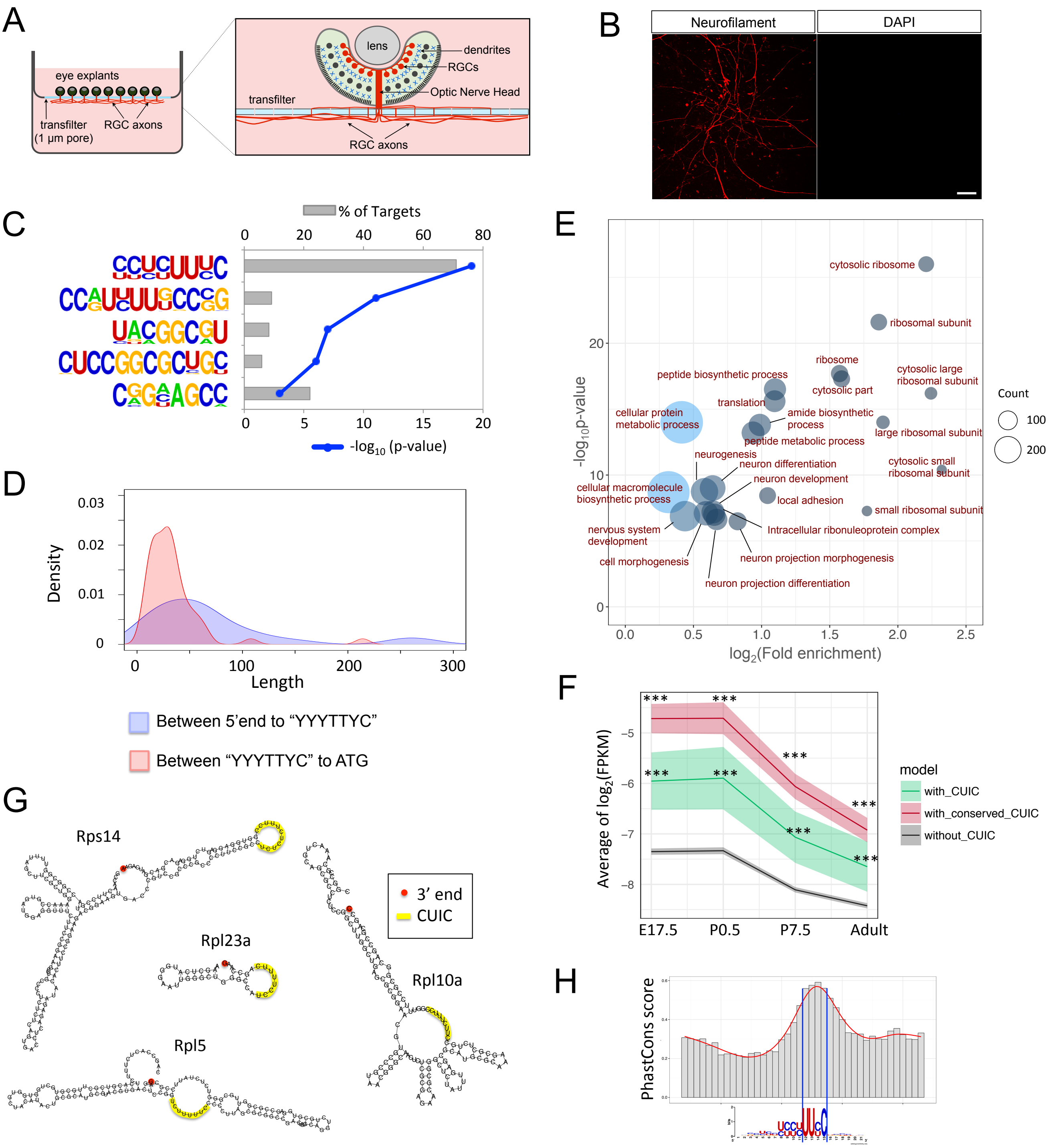

Figure S1, Related to Figure 1. (A) Diagram showing the physical separation of RGC axons from cell bodies in a Boyden chamber device. (B) Immunostaining images of pure axons, obtained with a Boyden chamber, with anti neurofilament-associated antigen antibody (3A10) and DAPI after the removal of cell bodies. Scale bar = 50µm. (C) A bar/line graph showing the result of *de novo* motif discovery for the 5' UTR of mouse RPs using HOMER. Grey bars show the percentage of motif-containing targets and the blue line represents the p-values. (D) Kernel density estimation of lengths between 5' end and the CUIC motif, and between the CUIC motif and the initiation codon (ATG). (E) Enrichment plot of GO terms (GOTERM_BP_5 and GOTERM_CC_5) of all CUIC-containing mouse genes, analyzed using DAVID. (F) A graph showing the average and 95% CI of the relative abundance (log2(FPKM)) of all genes with or without the CUIC motif in mouse RGC axons of 4 different developmental stages of mouse RGC axons (***p<0.001, Kolmogorov–Smirnov test, compared to "without CUIC"). (G) Examples of RNA secondary structures of CUIC containing 5' UTRs of several RPs. (H) Average interspecies conservation scores using PhastCons (mm10.60way.PhastCons) around the CUIC motif in mouse RP-coding mRNAs.

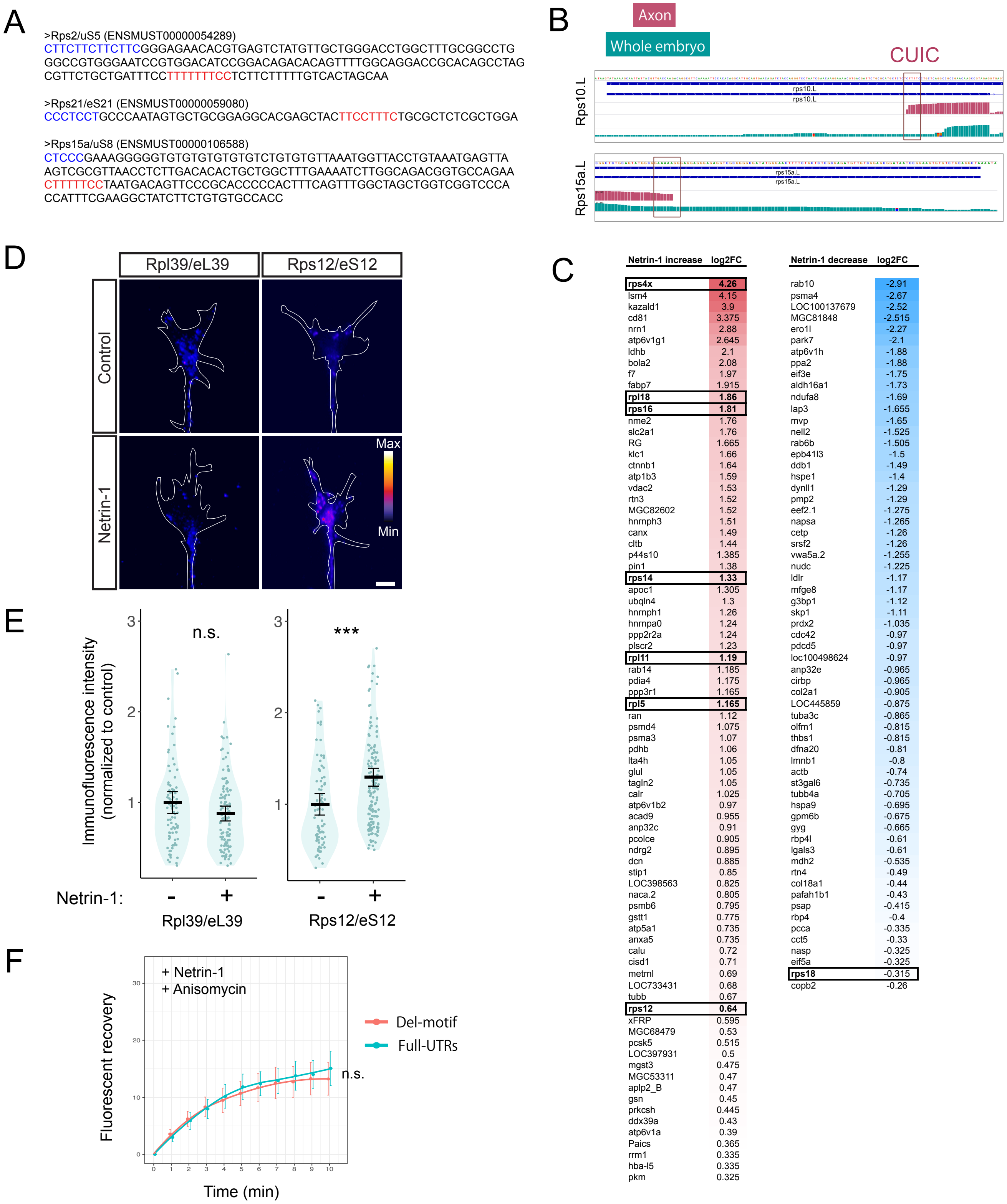

Figure S2, Related to Figure 2. (A) Mouse 5' UTR example sequences for several RP mRNAs containing CUIC (red) and 5'TOP (blue) motifs separately. (B) Genome browser view of coverage of RNA-seq reads on the 5'end of two RP-coding mRNAs showing 5' ends truncated close to the CUIC-motif in axons. (C) List of proteins whose translation in axons are significantly changed by Netrin-1 stimulation. RPs are highlighted in boxes. (D-E) Representative images (D) and qIF plots (E) for Rps12/eS12 and Rpl39/eL39 in axonal growth cones **with and without Netrin-1 following 30 min and 5 min stimulation, respectively (bars = average with 95% CI, Mann–Whitney U test, *** p≤0.001). Scale bar = 5µm. (F) Relative fluorescence recovery of indicated Venus reporter constructs after photobleaching (error bar = SEM) in RGC axons with anisomycin and Netrin-1 treatment.

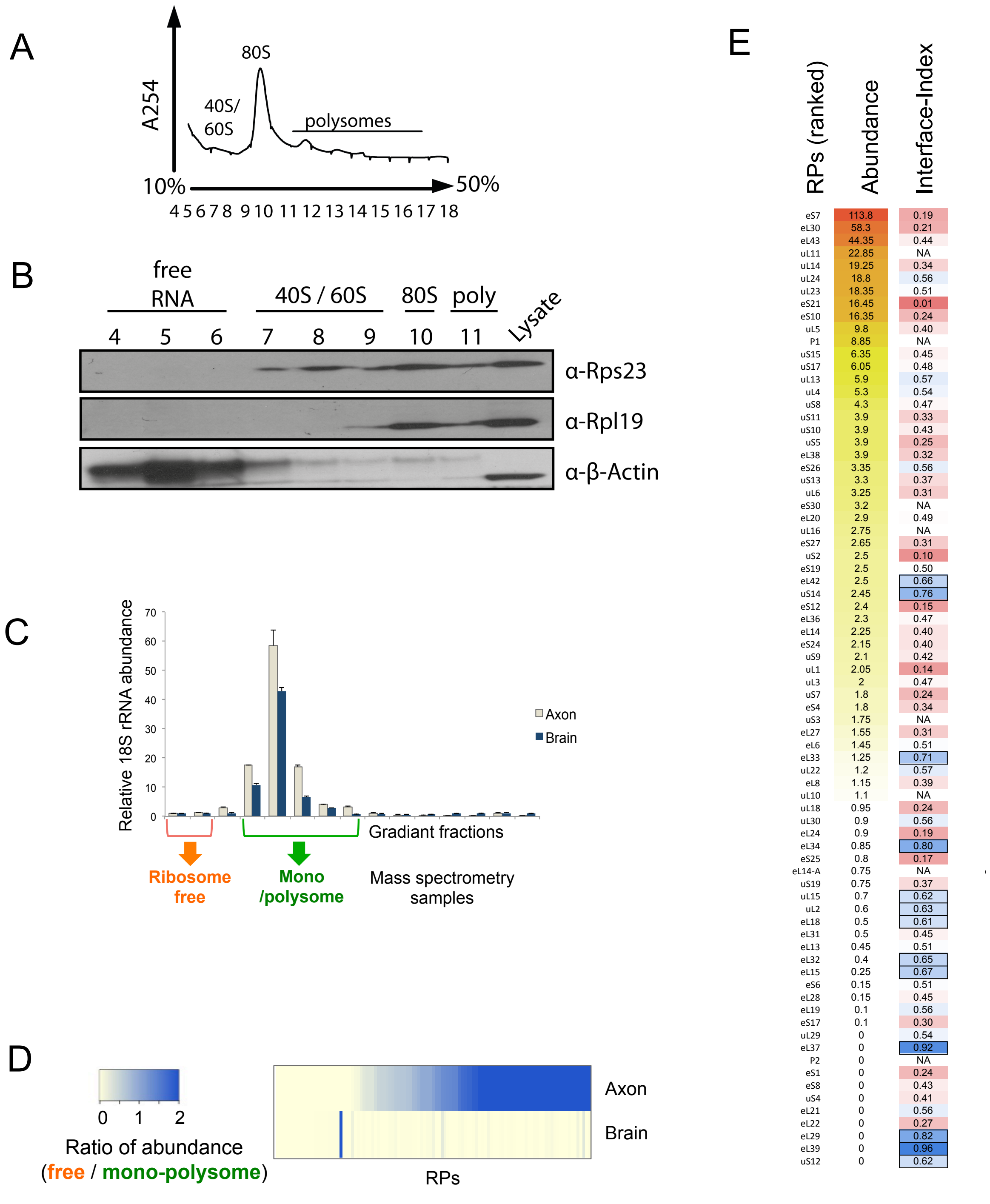

Figure S3, Related to Figure 3. (A-B) UV absorbance (A254) profile of sucrose density gradients (A) and Western blot analysis using antibodies against Rps23/uS12, Rpl19/eL19 and b-actin (B) for each fraction of the whole brain sample. (C) qRT-PCR detection of 18S rRNA in each fraction of the axon and whole brain sample after the sucrose density gradient fractionation. (D) Heat map showing ratio of RP abundance (free / mono-polysome) for the axon and whole brain sample (average of two biological replicates). (E) Heatmap with the ranking of abundance (LFQ abundance scores) of RPs detected in the ribosome free fraction of the axon sample, and their Interface Index scores (more blue = higher, more red = lower). High Interface-Indices (> 0.6, framed) were significantly depleted from the group of RPs with high abundance (LFQ abundance score > 1) in the axonal ribosome-free fraction (2.6 fold depletion, Fisher's exact test p = 0.0038)

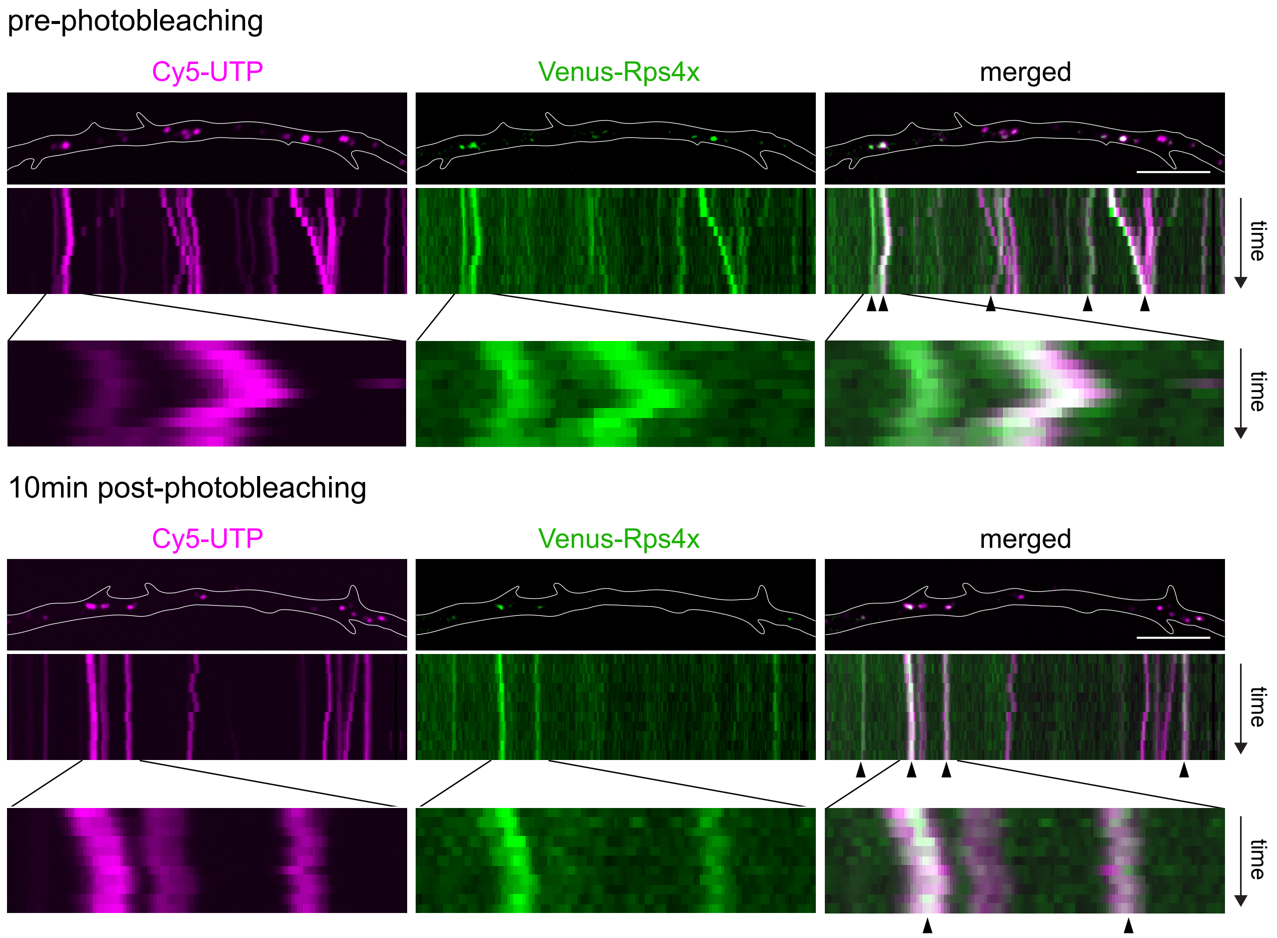

Figure S4, Related to Figure 4. Images and kymographs (30 sec) obtained from the movies of same axon in Figure 4D to show co-localization and co-movement between Cy5-UTP (magenta) and UTR-Rps4x/eS4-Venus fusion or UTR-Venus (green) reporter before and 10 min after photobleaching. Scale bar = 5µm.

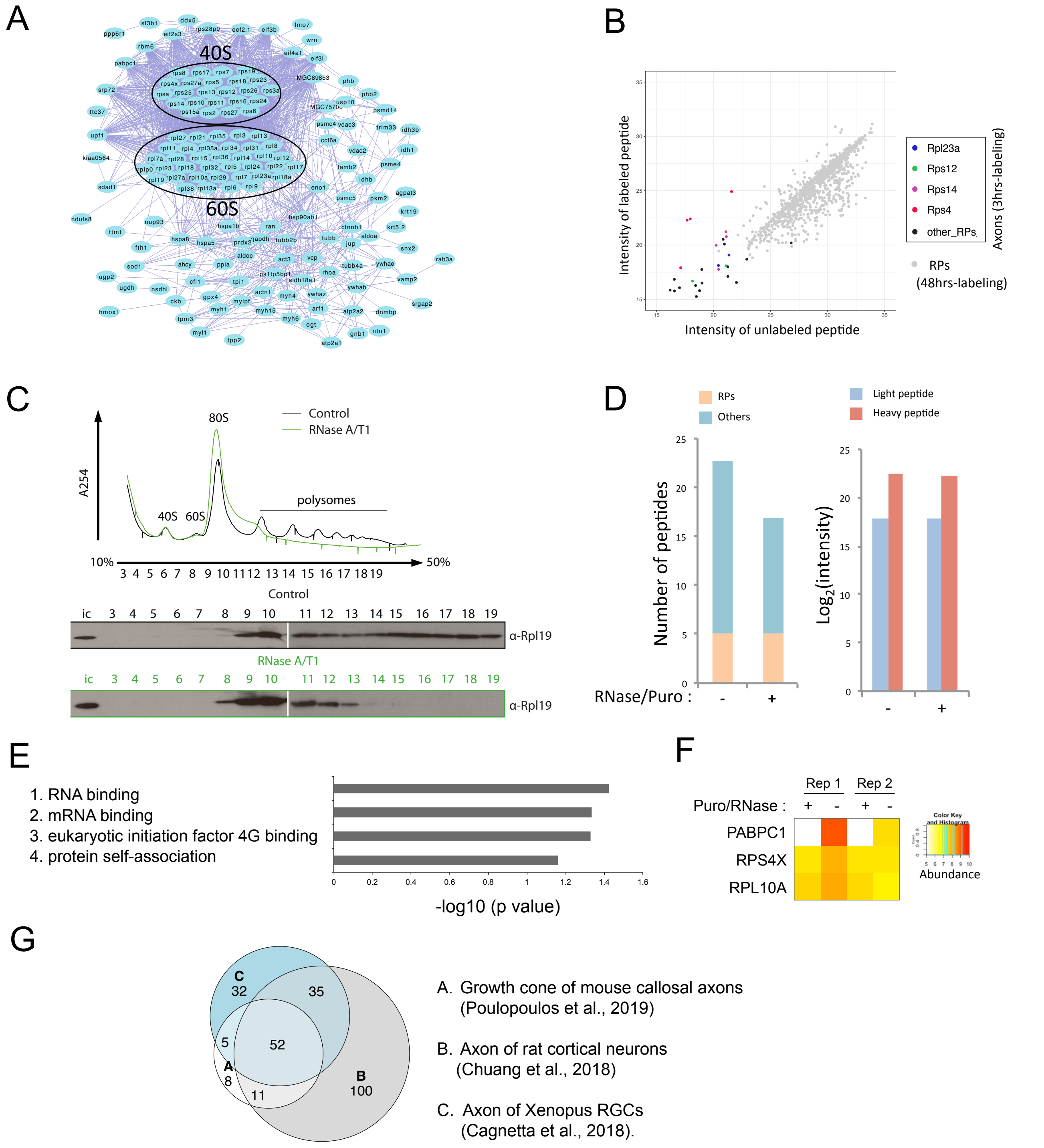

Figure S5, Related to Figure 5. (A) Gene network analysis of proteins detected in the axonal ribosome sample by MaxQuant. (B) Scatter plot showing abundance (log2 (intensity)) of labeled and unlabeled RP peptides detected in any of the biological replicates by MaxQuant. (C) UV absorbance (A254) profile and immunoblot detection of Rpl19/eL19 in each fraction of samples with and without RNase A/T1 treatment. (D) Left panel shows the average number of peptides (RPs: orange, other proteins: green) detected in the puromycin/RnaseA/T1-treated and untreated axonal ribosome sample. Right panel shows the comparison of the intensity of a peptide from Rps4x/eS4. (E) GO terms enriched in the group of which the LFQ intensities are significantly decreased in the brain ribosome sample by RNaseA/T1 treatment. (F) Heatmap showing the protein abundance (LFQ intensity) in the brain ribosome samples with or without the RNaseA/T1 treatment. (G) Venn diagram showing the overlapping of nucleolar/ribosomal assembly factors detected in axons among three different studies.

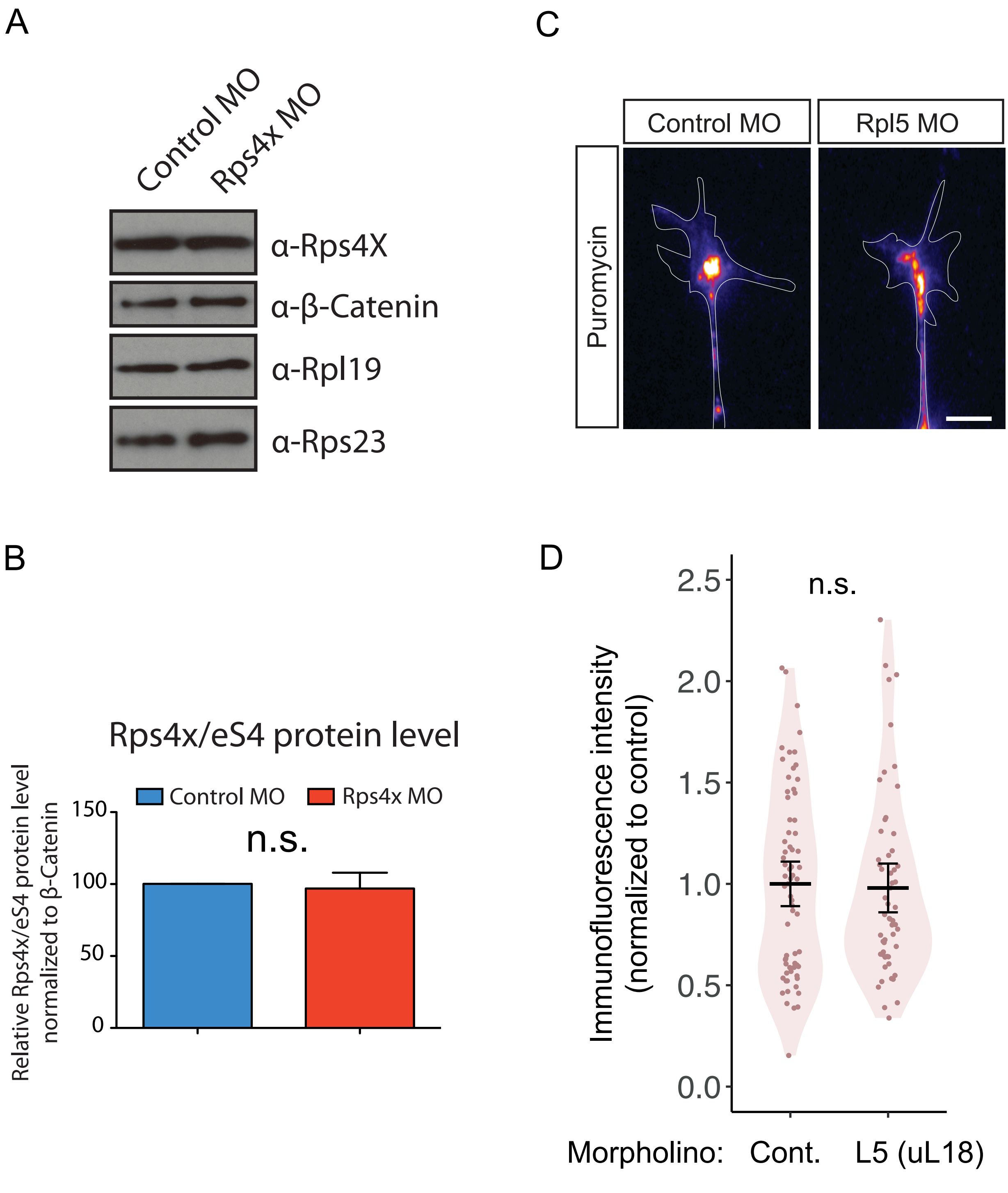

Figure S6, Related to Figure 6. (A) Western blots of protein samples isolated from the somal compartment from Control MO or Rps4x/eS4 MO treated samples. (B) Bar graph showing Western blot quantification of Rps4x/eS4 protein levels, normalized to β-Catenin protein levels. n = 3, error bars = SEM, Welch t-test (not significant). (C-D) Images (C) and plots (D) of average, 95% CI and distribution of normalized levels of puromycin incorporation in the axons treated with control morpholinos (Cont.) or with morpholinos against Rpl5/uL18, with Welch t-test (not significant).

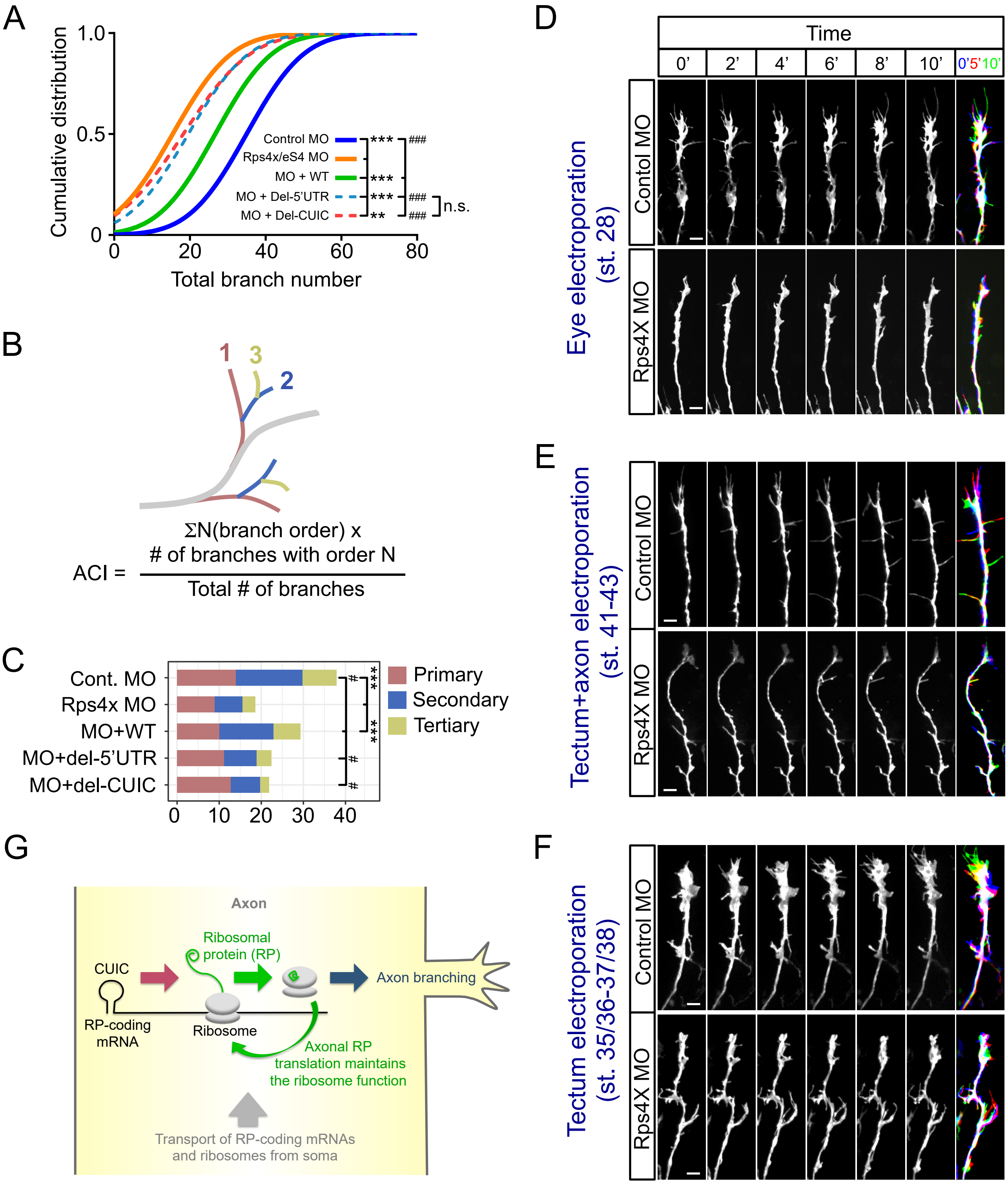

Figure S7, Related to Figure 7. (A) The cumulative distribution of total branch number (Extra sum-of-squares F test). (B) Formulation of axon complexity index (ACI). (C) A bar graph showing the number of branches in Control, Rps4x/eS4 morphants and Rps4x/eS4 morphants with different rescue constructs (primary: $F_{4,146}=5.3$, $p=0.0005$; secondary: $F_{4,146}=12.8$, $p<0.0001$; tertiary: $F_{4,146}=7.6$, $p<0.0001$; total: $F_{4,146}=11.14$, $p<0.0001$). (D-F)Time-lapse images of axonal branching in the tectum after eye electroporation (Whole cell KD, upper: stage 28) (D) and tectum electroporation at stages 41-43 (Axonal KD) (E) and at stage 35/36-37/38 (Tectum KD) (F). Scale bar = 20µm. (G) A model in which axonal RP synthesis supports the ribosome function and axon branching.
